# Classical Quantum Friction at Water–Carbon Interfaces

**DOI:** 10.1021/acs.nanolett.2c04187

**Published:** 2023-01-10

**Authors:** Anna T. Bui, Fabian L. Thiemann, Angelos Michaelides, Stephen J. Cox

**Affiliations:** † Yusuf Hamied Department of Chemistry, University of Cambridge, Lensfield Road, CambridgeCB2 1EW, United Kingdom; ‡ Thomas Young Centre, London Centre for Nanotechnology, and Department of Physics and Astronomy, University College London, Gower Street, LondonWC1E 6BT, United Kingdom; § Department of Chemical Engineering, Sargent Centre for Process Systems Engineering, Imperial College London, South Kensington Campus, LondonSW7 2AZ, United Kingdom

**Keywords:** liquid−solid friction, nanoscale water, liquid−solid interfaces, graphene, molecular dynamics

## Abstract

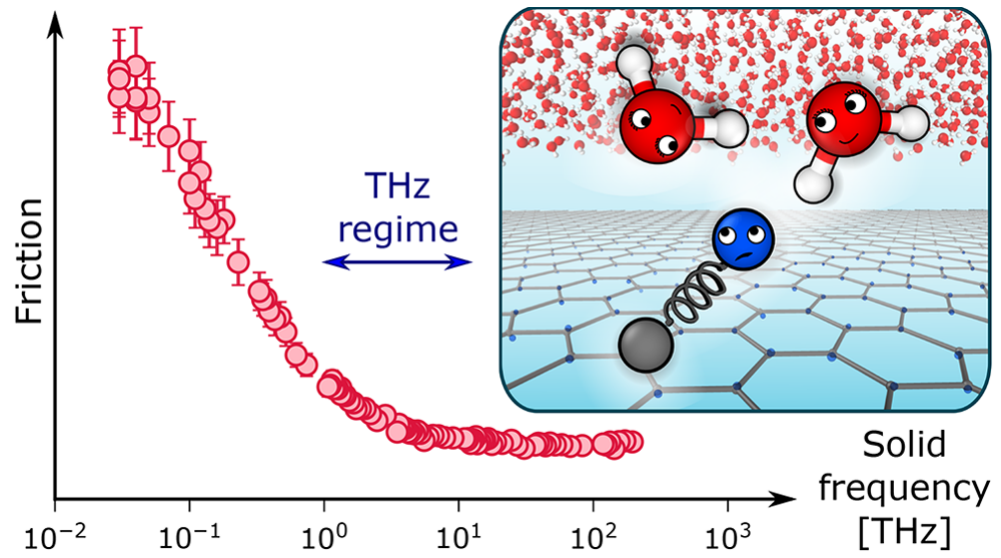

Friction at water–carbon interfaces remains a major puzzle with theories and simulations unable to explain experimental trends in nanoscale waterflow. A recent theoretical framework—quantum friction (QF)—proposes to resolve these experimental observations by considering nonadiabatic coupling between dielectric fluctuations in water and graphitic surfaces. Here, using a classical model that enables fine-tuning of the solid’s dielectric spectrum, we provide evidence from simulations in general support of QF. In particular, as features in the solid’s dielectric spectrum begin to overlap with water’s librational and Debye modes, we find an increase in friction in line with that proposed by QF. At the microscopic level, we find that this contribution to friction manifests more distinctly in the dynamics of the solid’s charge density than that of water. Our findings suggest that experimental signatures of QF may be more pronounced in the solid’s response rather than liquid water’s.

Recent advances in nanofluidics^1,2^ show great promise for membrane-based desalination technologies^3−5^ and energy harvesting applications.^6−11^ Owing to the relative ease of fabricating carbon-based nanostructures, a feature common to many of these technologies is the presence of extended interfaces between liquid water and carbon. Despite significant research effort, there are still major gaps^12−15^ in our understanding of water at graphitic surfaces. Of particular curiosity, experiments have found that friction of water on carbon surfaces is ultralow compared to other two-dimensional materials.^16−20^ In addition, the friction of water is much higher on multilayer graphite^21−23^ than monolayer graphene^24^ and a peculiar radius dependence in multiwalled carbon nanotubes^25,26^ is observed. Reproducing these observations has so far remained beyond the realms of molecular simulations,^27−30^ even with highly accurate interatomic potentials.^31^ Consequently, these observations cannot be explained by the traditional “surface roughness” approach^32,33^ that underpins much of our understanding of friction at liquid–solid interfaces.

A recent theoretical study^34^ by Kavokine et al. has sought to explain the differences in friction at graphene vs graphite by accounting for coupling between collective charge excitations of the liquid and the dynamics of electrons in the carbon substrate. In this framework of “quantum friction” (QF), friction of water on graphite is argued to be larger than that on graphene due to the presence of a dispersionless surface plasmon mode in graphite^35−37^ that overlaps with liquid water’s terahertz (THz) dielectric fluctuations.^38−40^ The purpose of the present article is to explore QF with molecular simulations.

Such coupling between electronic motion in the solid and charge density fluctuations in the liquid is an effect beyond the Born–Oppenheimer approximation.^41^ While simulation schemes to account for such nonadiabatic dynamics (“electronic friction”) exist,^42−44^ they rely on the accurate construction of a (3*N* × 3*N*) friction tensor, where *N* is the total number of atoms explicitly considered in the dynamics. So far, their application has been limited to single gas-phase molecules on metal surfaces,^45−48^ where the friction coefficient on each atom can be well-approximated to depend only on the solid electron density locally.^49,50^ The low-frequency dielectric modes of water, which are essential to the description of QF, however, are inherently collective in nature, prohibiting the application of these sophisticated methods at present.

While accurately accounting for nonadiabatic electronic motion is computationally challenging, the low-frequency dielectric response of water is reasonably well captured by simple point charge models.^51,52^ In this article, we therefore focus on this aspect of QF—that dissipative friction forces are mediated through a complex interplay of charge density fluctuations—which is more amenable to classical molecular dynamics (MD) simulations. By extending a standard treatment for polarizability in graphene such that its dielectric fluctuations can be precisely controlled, we will show that coupling between charge density fluctuations in the solid and liquid increases friction in line with the predictions of QF. Also similar to QF, this additional contribution is distinct from the typical surface roughness picture for friction. The insights afforded by our simulations suggest that microscopic signatures of QF manifest more distinctly in the dynamics of the solid’s dielectric fluctuations rather than in the structure or dynamics of liquid water.

## Model of the Liquid–Solid Interface

**Figure 1:**
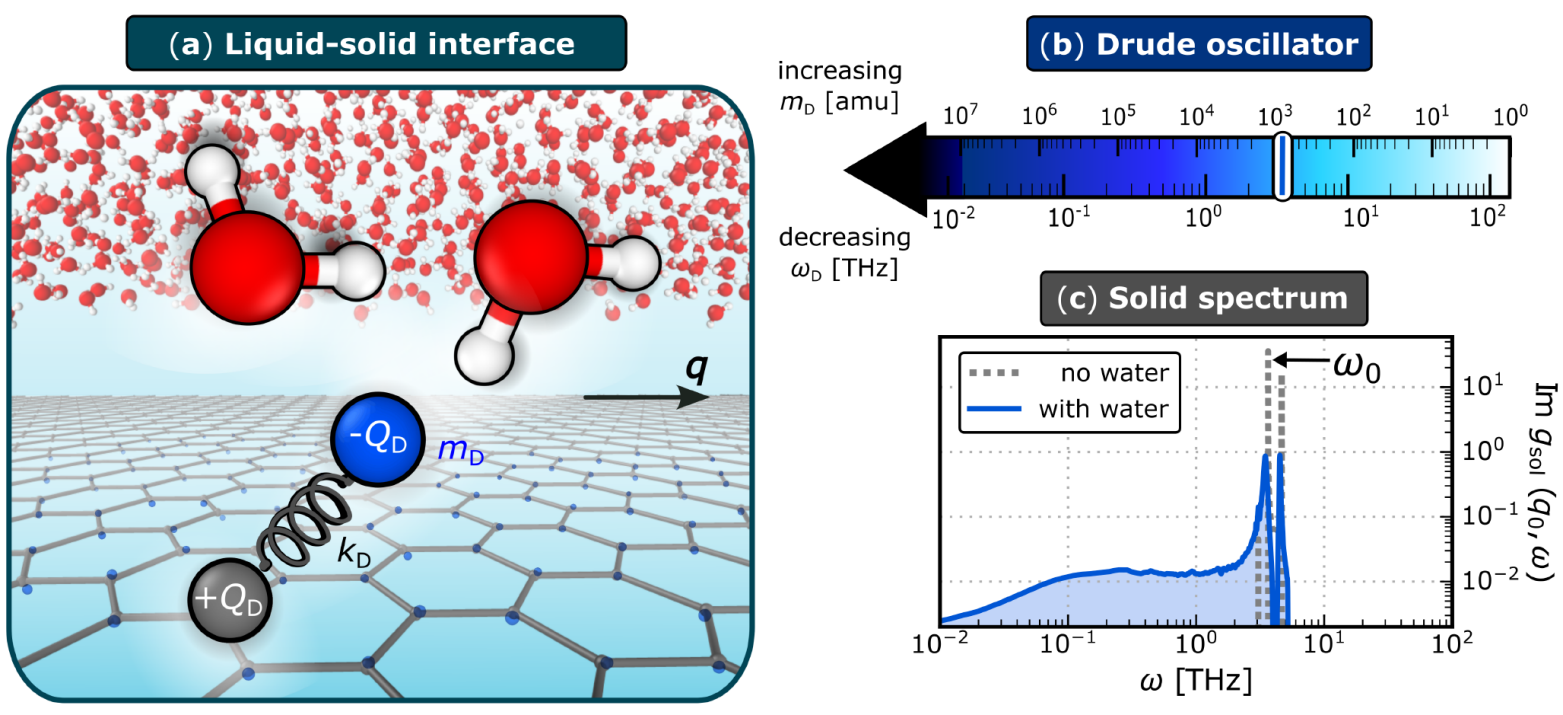
Model of the liquid–solid interface. (a) Schematic illustrating the interface of a film of water on a graphene sheet. The graphene’s charge density is described by a classical Drude oscillator model: each C atom carries a charge +*Q*_D_ and is attached to a fictitious Drude particle of mass *m*_D_ and charge −*Q*_D_ via a harmonic spring with force constant *k*_D_. O, H, C atoms and Drude particles are in red, white, gray, and blue, respectively. For clarity, only one Drude particle is highlighted and its displacement from the C atom is exaggerated (see the Supporting Information for the actual distribution). The characteristic frequency of the Drude oscillators,  is controlled by varying *m*_D_, as indicated by the color bar in b. (c) In the absence of water, the surface response function of the solid, *g*_sol_(*q*_0_, ω) (shown for ω_D_ = 3.3 THz), is dominated by two peaks, with ω_0_ describing the position of the lower frequency peak. In the present case, ω_0_ ≈ 3.5 THz ≈ ω_D_, indicating a weak coupling between the Drude oscillators. With water present, these peaks are broadened.

The system we consider consists of a thin film of water on a frozen flat graphene sheet, as shown schematically in Figure 1a. To model water, we use the SPC/E model,^53^ which reasonably captures both the librational modes (hindered molecular rotations) as a sharp peak at ω_lib_ ≈ 20 THz, and the Debye modes (hindered molecular translations) as a broad feature spanning ∼10^–2^–10^1^ THz.^51,52^ Water–carbon interactions are modeled with a Lennard-Jones potential that reproduces the contact angle of water droplets on graphitic surfaces;^54^ while such a potential captures the essential features of surface roughness contributions to friction, it lacks any dielectric response. For each carbon center, we therefore also ascribe a charge +*Q*_D_ and attach to it, via a harmonic spring with force constant *k*_D_, a “Drude particle” of mass *m*_D_ and charge −*Q*_D_. This classical Drude oscillator model is a common approach for modeling electronic polarizability^55^ and introduces electrostatic interactions between both the water film and the substrate, and the substrate with itself. In the absence of water, the graphene sheet can be considered a set of weakly interacting harmonic oscillators (see the Supporting Information).

To parametrize the model, we set *Q*_D_ = 1.852 *e* and *k*_D_ = 4184 kJ mol^–1^ Å^–2^, which have been shown to recover the polarizability tensor of a periodic graphene lattice.^56^ In usual treatments of electronic polarizability, one follows a Car–Parrinello-like scheme,^57^ whereby *m*_D_ is chosen to be sufficiently small to ensure adiabatic separation of the Drude and nuclear (in this case, water) motions. Here, we are inspired by the fact that, even for bulk systems, increasing *m*_D_ leads to nuclear motion experiencing drag forces.^58^ We therefore treat *m*_D_ as a free parameter that tunes the frequency  of an individual oscillator, anticipating that this may lead to an increase in friction at the liquid–solid interface. However, the details of how friction may vary with ω_D_ are not *a priori* obvious. In practice, we choose 1 ≲ *m*_D_/amu ≲10^7^, such that 10^–2^ ≲ ω_D_/THz ≲ 10^2^ as indicated in Figure 1b. Importantly, changing *m*_D_ in this manner does not affect the system’s static equilibrium properties.

In QF, significant overlap between the substrate and water is due to a dispersionless plasmon mode present in graphite but not graphene. While we cannot reasonably expect the classical Drude model to faithfully describe this plasmonic behavior, we can ask a more general question concerning how friction is affected when the substrate and fluid spectra overlap significantly. This question can readily be addressed by tuning *m*_D_, as we describe above, without the need to introduce multilayer graphite. For simplicity, and ease of comparison between systems, we therefore employ a single graphene sheet in all simulations.

Overall, our model describes two fluctuating charge densities, *n*_wat_(***r***, *t*) of the water and *n*_sol_(***r***, *t*) of the solid, originating from the collective motion of water molecules and Drude oscillators, respectively, at position ***r*** and time *t*. The total charges of both the water and the solid are strictly conserved. It will be convenient to characterize these charge distributions by their surface response functions,^34,59^ e.g., for water
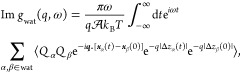
1where  is the interfacial lateral area, *k*_B_ is Boltzmann’s constant, *T* is the temperature, ***q*** is a wavevector parallel to the surface, and *Q*_α_ is the charge on atom α whose position in the plane of the graphene sheet at time *t* is ***x***_α_(*t*) with vertical coordinate *z*_α_(*t*) = *z*_0_ + Δ*z*_α_(*t*), where *z*_0_ defines a plane between the carbon atoms and the water contact layer. The surface response function of the solid, *g*_sol_(*q*, ω), is similarly defined.

In Figure 1c, we present *g*_sol_(*q*_0_, ω) for *m*_D_ = 10^3^ amu both in the absence and presence of water, where *q*_0_ = 2π/*L*_*x*_ ≈ 0.25 Å^–1^ corresponds to a low wavevector accessible in the simulation box. In the absence of water, *g*_sol_(*q*_0_, ω) exhibits two dominant peaks (see the Supporting Information). We will focus on the lower frequency peak, whose position we take to be ω_0_. As ω_0_ ≈ ω_D_, it is appropriate to consider the graphene sheet as a set of weakly coupled harmonic oscillators (see the Supporting Information). In the presence of water, both of these peaks are broadened, and we also see the emergence of a broad feature at low frequencies. We will discuss the implication of these observations in the context of friction below. Further technical details of the model, simulation setup, precise definitions of computed quantities and additional tests for the sensitivity of our results to the choice of simulation settings are given in the Supporting Information.

## Friction at the Water–Carbon Interface Depends Sensitively on ω_0_

We proceed to explore how the features of *g*_sol_(*q*_0_, ω) affect friction at the interface. For each value of *m*_D_, we perform equilibrium MD simulations to extract the liquid–solid friction coefficient λ from the well-established Green–Kubo relationship:^32,60^
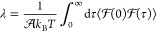
2where  is the total force acting on the liquid along a Cartesian direction lateral to the graphene sheet at time τ and ⟨···⟩ indicates an ensemble average.

**Figure 2:**
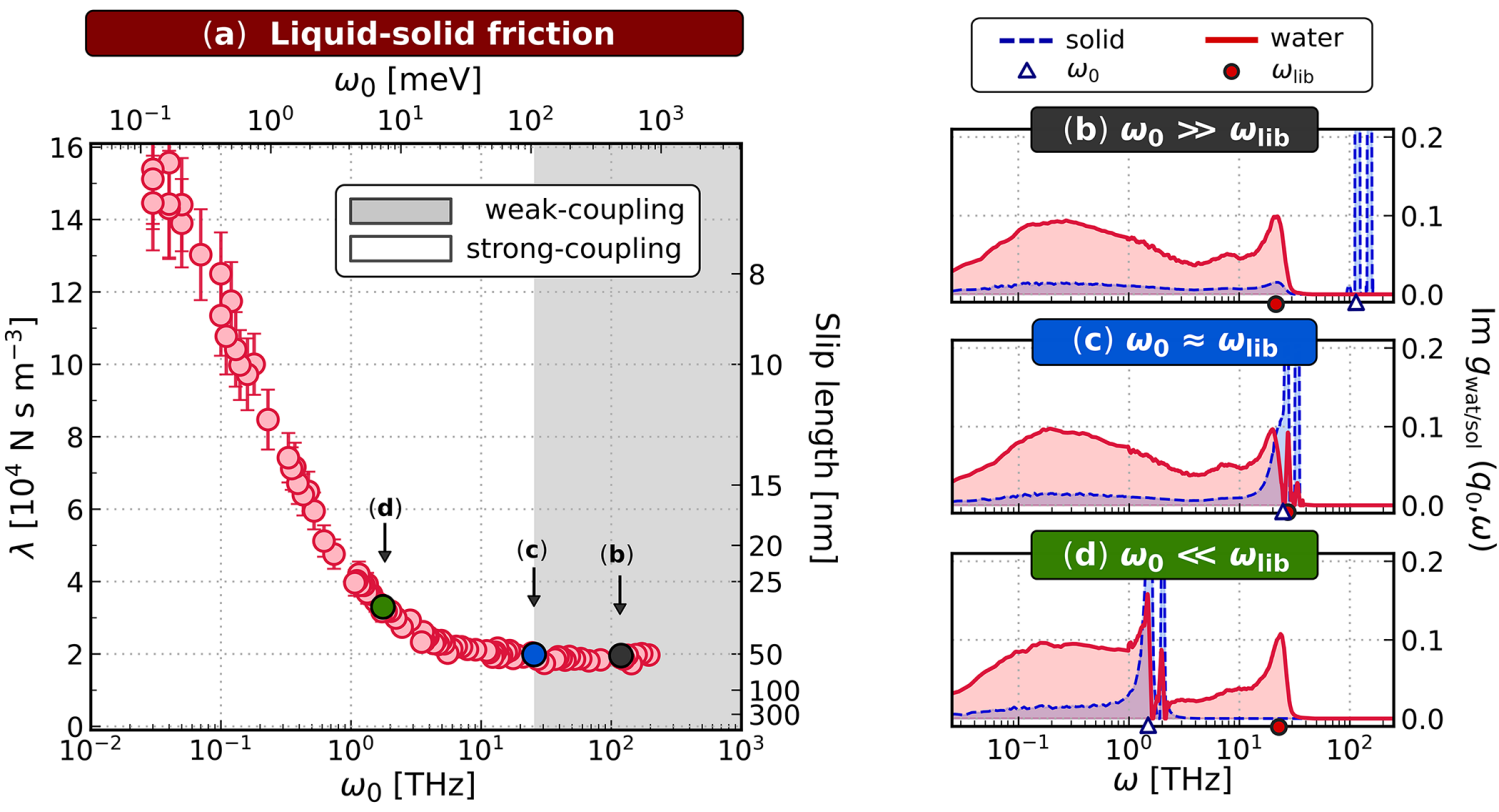
Friction increases as dielectric fluctuations begin to overlap. (a) The liquid–solid friction coefficient λ is shown against ω_0_. The slip length is given as *b* = η/λ where η is the viscosity of water. Statistical errors are obtained from block-averaging. Two regimes are indicated: weak-coupling (shaded gray) where λ remains roughly constant; and strong-coupling (not shaded) where λ increases with decreasing ω_0_. (b–d) *g*_wat_(*q*_0_, ω) and *g*_sol_(*q*_0_, ω) are shown for three representative cases ω_0_ ≫ ω_lib_, ω_0_ ≈ ω_lib_, and ω_0_ ≪ ω_lib_, respectively. We see that the increase in λ coincides with ω_0_ ≲ ω_lib_ ≈ 20 THz. In addition, when the overlap between the spectra is significant, the dominant features of *g*_sol_(*q*_0_, ω) are broadened, and *g*_wat_(*q*_0_, ω) is perturbed. The boundary between the regimes is approximate.

In Figure 2a, we show the dependence of λ on ω_0_ in the range 10^–2^–10^2^ THz from a total of 97 simulations. Overall, as ω_0_ decreases, λ stays constant until ω_0_ ≈ ω_lib_ ≈ 20 THz, whereupon further decreasing ω_0_ leads to a significant increase in λ. To rationalize this observation, we inspect *g*_wat_(*q*_0_, ω) and *g*_sol_(*q*_0_, ω), as shown in Figure 2b–d, for three representative cases. Based on the relative positions of ω_0_ (the principal frequency of the solid) and ω_lib_ (liquid water’s librational frequency), we separate the liquid–solid frictional response into two regimes:

(i) Weak-coupling regime: When ω_0_ ≳ ω_lib_, the friction coefficient remains roughly constant at λ ≈ 1.9 × 10^4^ N s m^–3^. This value agrees well with previous simulations of water on graphitic surfaces.^27,31,61−64^ In this regime, there is a large separation of time scales between the dielectric modes of water and the substrate. As a result, there is little overlap between *g*_sol_(*q*_0_, ω) and *g*_wat_(*q*_0_, ω), as seen in Figure 2b, and water’s dynamics are largely unaffected by varying ω_0_. The motions of the Drude oscillators and the water are not strongly coupled.
(ii) Strong-coupling regime: When ω_0_ ≲ ω_lib_, hydrodynamic friction increases as ω_0_ decreases, reaching λ ≈ 15 × 10^4^ N s m^–3^ for ω_0_ ≈ 0.03 THz. This change in friction of just over 1 order of magnitude would lead to a significant change in the corresponding slip length from ∼60 nm to ∼7 nm. For comparison, experiments have reported water slippage in the range of 0–200 nm on graphene^24^ and 8–13 nm on graphite.^21−23^ In this regime, there is no longer a large separation in time scales between the Drude oscillators and water’s dielectric modes. Consequently, as seen in Figure 2c, d, *g*_sol_(*q*_0_, ω) now overlaps strongly with water’s librational and Debye modes, causing changes in *g*_wat_(*q*_0_, ω) that reflect the dominant features of *g*_sol_(*q*_0_, ω). The onset of this regime is further supported by the broadening of the dominant peaks in *g*_sol_(*q*_0_, ω) and changes in the spectrum of the lateral force on the liquid (see the Supporting Information). We conclude that the increase in friction in this strong-coupling regime is indeed due to coupling of the dielectric modes in the water and the substrate.

To test the sensitivity of this separation into strong- and weak-coupling regimes to the details of the system, we have also performed simulations with different harmonic potentials for the Drude oscillators and a flexible water model (see the Supporting Information). While differences in the absolute values of λ are expected, and indeed observed, the increase of λ for ω_0_ ≲ ω_lib_ is robust.

## Comparing Molecular Simulations with Quantum Friction Theory

**Figure 3:**
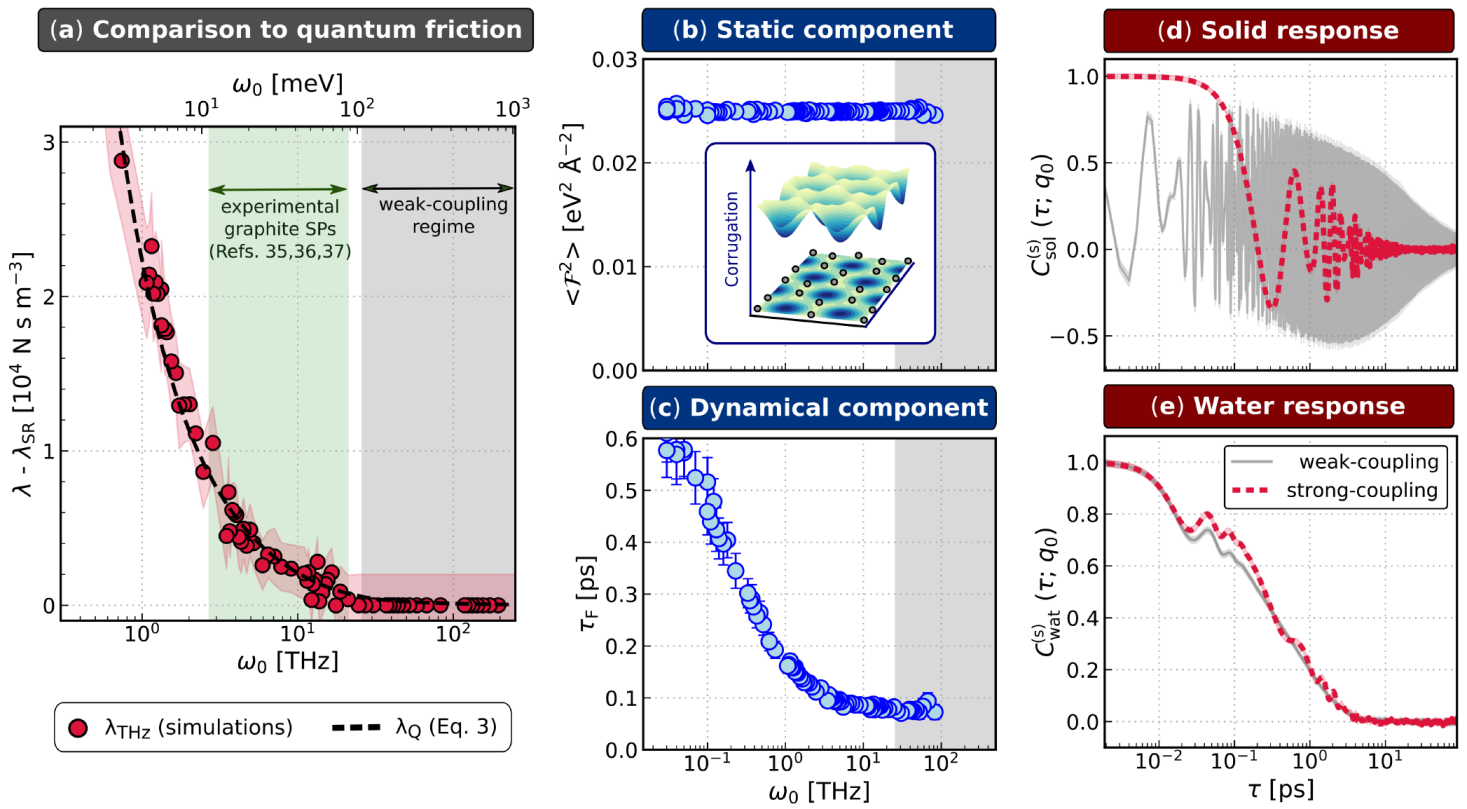
Microscopic signatures of quantum friction. (a) The prediction of QF (λ_Q_, eq 3) well describes λ_THz_ obtained directly from molecular simulations. The shaded red region indicates the standard deviation from block-averaging. A range of frequencies for experimental surface plasmons (SPs) in graphite is indicated by the green shaded region. (b) The static component of the total friction  is essentially independent of ω_0_, and therefore, all liquid–solid interfaces simulated have the same free energy surface shown in the inset (see the Supporting Information for more details). (c) In contrast, the dynamical component τ_F_ fully captures the dependence of λ on ω_0_. Surface charge density correlation functions are shown for representative cases from the weak-coupling (ω_0_ ≈ 100 THz) and strong-coupling (ω_0_ ≈ 1 THz) regimes for (d) the solid and (e) water.  decays much more quickly in the strong-coupling regime, while changes to  are much less pronounced. The legend in panel (e) also applies to panel (d).

Before further analysis, it is useful to make a comparison of our simulation results to QF theory.^34^ Kavokine et al. separated the liquid–solid friction into λ = λ_SR_ + λ_Q_, where λ_SR_ is the classical surface roughness contribution and
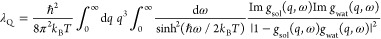
3is the contribution from quantum friction. In our simulations, changing *m*_D_ does not affect static equilibrium properties such as surface roughness (see the Supporting Information). In analogy to QF, then, we also decompose the friction coefficient from simulation as λ(ω_0_) = λ_SR_ + λ_THz_(ω_0_), where λ_THz_ originates from the coupling of charge density fluctuations in the THz regime.^65^ We can obtain approximate expressions for *g*_sol_(*q*, ω) and *g*_wat_(*q*, ω) appropriate for our simulations. As detailed in the Supporting Information, for *g*_wat_(*q*, ω) we use a parametrization specified in ref (34). for SPC/E in contact with graphene/graphite. For *g*_sol_(*q*, ω), we parametrize a semiclassical Drude model for the surface plasmon^66^ to roughly capture the intensity and width of the principal peak of *g*_sol_(*q*, ω) observed in our simulations. In Figure 3a, we compare λ_Q_ given by eq 3 using these suitably parametrized surface response functions to λ_THz_ obtained directly from our simulations. The excellent agreement between the simulation result and eq 3 provides strong support for the theory of quantum friction outlined in ref (34).

## Microscopic Signatures of Quantum Friction Manifest in the Solid, Not the Liquid

A major advantage of performing molecular simulations is the insight they can provide at the microscopic scale. While treating electronic motion as a set of weakly coupled classical Drude oscillators lacks any explicit treatment of quantum mechanical effects, the good agreement between this classical model and QF reinforces the importance of water’s low-frequency dielectric modes in any potential nonadiabatic contributions to friction at water–carbon interfaces.

Going further, we follow ref (62) by disentangling the origin of the friction at the interface by reformulating eq 2 as , such that the mean-squared force  and force decorrelation time τ_F_ quantify static and dynamical components, respectively. As seen in Figure 3b, the static component remains essentially constant across the entire range of ω_0_ explored. This implies that the water molecules experience the same free energy surface at the interface, an example of which is shown in Figure 3b, inset, independent of ω_0_ (see the Supporting Information). This confirms that the physical origins of λ_THz_ are not captured by the corrugation of the free energy surface that has been widely used to account for the curvature dependence of friction in CNTs^27,31^ and certain differences in hydrodynamic slippage at different materials.^31,33,61,62,67^ Instead, the nature of λ_THz_ is entirely dynamical, with the dependence of τ_F_ on ω_0_ accounting entirely for the increase in λ_THz_, as seen in Figure 3c.

The above analysis demonstrates that microscopic signatures of nonadiabatic friction should manifest in dynamical rather than static properties of the system. We therefore consider the surface charge densities, e.g., for water
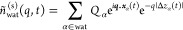
and inspect their autocorrelation functions  and ; these are presented in Figure 3d and e, respectively, for *q* = *q*_0_. While small changes in  are observed between the weak- and strong-coupling regimes, the impact on  is much more pronounced. For the water film, we have also probed molecular reorientation and hydrogen bond relaxations, and found that these are barely affected between the two regimes. This suggests that quantum friction is unlikely to have a significant impact on water’s local dynamical properties.

We attribute these contrasting behaviors of the liquid and the solid to the rigidity of water’s hydrogen-bond network, which lacks a clear counterpart from the perspective of the Drude oscillators. In fact, it is even useful to simply compare the relative magnitude of the dipoles for a single water molecule μ_wat_ and a Drude oscillator μ_D_. With our simple point charge model, we have ⟨μ_wat_⟩ = 2.351 D, while . Thus, while the water molecules only feel the presence of the Drude oscillators as a small perturbation relative to their intermolecular interactions, the Drude oscillators feel the impact of the water molecules much more strongly. We speculate that this conclusion also applies to cases where electronic degrees of freedom have been accurately accounted for.

In summary, by using a simple model of charge density fluctuations in a carbon substrate in which we can finely tune the surface response function of the substrate, we find increases in interfacial friction in line with those suggested by a recent theory of quantum friction. We see that the friction increases once the principal peak in the substrate’s surface response function overlaps with features in water’s surface response function arising from its librational and Debye modes. We show that this extra contribution to the friction is entirely dynamical in its origin, with static equilibrium properties apparently indifferent to the degree of coupling between the water and the substrate. The insights provided by our molecular simulations reveal that the increase in friction manifests at the microscopic scale as a pronounced change in the relaxation of the substrate’s dielectric modes, with relatively little impact on the behavior of water.

Our model, while able to provide a proof of concept for QF, does not aim to be a rigorous description of water on graphite. We have considered a static graphene sheet, which precludes any role that phonon modes might play.^68−72^ Any changes in surface roughness upon changing from single to multilayer systems have also not been accounted for. Going forward, it will be essential to explore how these factors affect both the surface roughness and charge density coupling contributions to friction. Advances in simulations of nonadiabatic effects^43,73,74^ to accurately describe the solid’s electronic excitations in response to collective fluctuations in the liquid will also be a welcome development. An obvious limitation of the present model is that it is restricted to describing the substrate as a dielectric, rather than a conductor (or semimetal). In principle, extending the current methodology to classical representations of metallic substrates^75,76^ should be relatively straightforward.

Despite its simplifications, our model captures the increase in the interfacial friction when there is an overlap in the dielectric spectra of the liquid and the solid. It is important to stress that this principle can be generalized to the interfaces of any combination of polar liquid and solid. Since the terahertz densities of state of a liquid can be reasonably described in simulations, our model opens up the possibility to predict whether different liquids^77,78^ also show a significant QF component. In addition to providing early evidence from simulations in general support of QF theory, our results suggest a potentially useful strategy for experimental verification. Specifically, the apparent asymmetry between the impact on water and the substrate suggests it may be advantageous to focus experimental efforts on spectroscopies that probe the substrate’s electronic response,^79^ rather than seeking hallmarks in the structure or dynamics of the liquid.

## Data Availability

The data that support the findings of this study are openly available at the University of Cambridge Data Repository at 10.17863/CAM.89536.

## Supporting Information Available

Model and simulation details; precise definitions and computational details of the quantities presented in the article; further analysis on the coupling of the liquid and solid charge densities; sensitivity of the results to certain aspects of the simulations and the model; detailed comparison to quantum friction theory and analyses on additional properties of the interface (PDF)
