# Supplementary material for: Classical Quantum Friction at Water–Carbon Interfaces
Source: Nano Lett. 2023 Jan 10;23(2):580–7. doi: 10.1021/acs.nanolett.2c04187 (PMC9881168; doi:10.1021/acs.nanolett.2c04187)
Supplement: Supplementary file 1 — nl2c04187_si_001.pdf [file nl2c04187_si_001.pdf]

Supporting Information for: Classical Quantum Friction at Water–Carbon Interfaces

Anna T. Bui,[1] Fabian L. Thiemann,[2,1,3] Angelos Michaelides,[1] and Stephen J. Cox[1]

[1] *Yusuf Hamied Department of Chemistry, University of Cambridge, Lensfield Road, Cambridge, CB2 1EW, United Kingdom*

[2] *Thomas Young Centre, London Centre for Nanotechnology, and Department of Physics and Astronomy, University College London, Gower Street, London, WC1E 6BT, United Kingdom*

[3] *Department of Chemical Engineering, Sargent Centre for Process Systems Engineering, Imperial College London, South Kensington Campus, London, SW7 2AZ, United Kingdom*

(Dated: January 9, 2023)

This supplementary information provides additional details on the results presented in the main article. This includes: the model and simulation details; precise definitions and computational details of the quantities presented in the article; further analysis on the coupling of the liquid and solid charge densities; sensitivity of the results to certain aspects of the simulations and the model; comparison to quantum friction theory and analyses on additional properties of the interface.

CONTENTS

S1. Classical molecular dynamics simulation details S3
- S1.1. Model description S3
- S1.2. System set-up S4
- S1.3. Simulation details S5

S2. Computation of properties S6
- S2.1. Friction coefficient S6
- S2.2. Surface response function S6
- S2.3. Surface charge density autocorrelation function S7

S3. Sensitivity of the friction coefficient S8
- S3.1. Convergence of the Green–Kubo friction coefficient S8
- S3.2. System size S9
- S3.3. Simulation time S9
- S3.4. Time-step S10
- S3.5. Thermostats S11
- S3.6. Liquid film thickness S12
- S3.7. Electrostatic boundary conditions S13

S4. Sensitivity of the dependence of friction on solid charge density frequency S14
- S4.1. Varying the Drude charge S14
- S4.2. Varying the spring constant S15
- S4.3. Flexible water model S15
- S4.4. Phonon contribution S16

S5. Coupling of charge densities S18
- S5.1. Solid surface response function in the absence of water S18
- S5.2. Solid surface response function in the presence of water S19
- S5.3. Water surface response function S20
- S5.4. Force spectra S21

S6. Comparison to quantum friction theory S23
- S6.1. Quantum friction formula S23
- S6.2. Water surface response S23

S6.3. Solid surface response with reparameterized Drude model S23
S6.4. Comparison of the quantum friction coefficient S24
S6.5. Sensitivity of the dependence of quantum friction on the solid frequency S25

S7. Additional properties of the interface S26
S7.1. Static properties S26
S7.2. Charge density relaxation in the water film S27
S7.3. Other dynamical properties S28

References S30

S1. CLASSICAL MOLECULAR DYNAMICS SIMULATION DETAILS

S1.1. Model description

We consider a system of a film of liquid water on a flat graphene sheet as described in the main article. Liquid water can be modeled by rigid simple point charge models with potential energy functions of the form

$$\mathcal{U}_{\mathrm{wat}}(\boldsymbol{R}_{\mathrm{wat}}^N) = \sum_{i<j}^{N} u_{\mathrm{LJ}}(|\boldsymbol{r}_{\mathrm{O},i} - \boldsymbol{r}_{\mathrm{O},j}|) + \sum_{i<j}^{N} \sum_{\alpha,\beta} \frac{Q_{\alpha,i} Q_{\beta,j}}{|\boldsymbol{r}_{\alpha,i} - \boldsymbol{r}_{\beta,j}|}, \tag{S1}$$

where $\boldsymbol{R}_{\mathrm{wat}}^N$ denotes the set of atomic positions for a configuration of N water molecules, $\boldsymbol{r}_{\mathrm{O},i}$ denotes position of the oxygen atom on water molecule i and $Q_{\alpha,i}$ is the charge of site α located at position $\boldsymbol{r}_{\alpha,i}$. The first set of sums in Eq. S1 captures short-ranged repulsion and non-electrostatic "long-ranged" attraction between water molecules with the usual Lennard-Jones 12-6 potential

$$u_{\mathrm{LJ}}(r) = 4\epsilon \left[\left(\frac{\sigma}{r}\right)^{12} - \left(\frac{\sigma}{r}\right)^{6} \right], \tag{S2}$$

which is parameterized by an energy scale ϵ and a length scale σ. The second set of sums in Eq. S1 describes electrostatic interactions. Here, we adopt a unit system for electrostatics in which $4\pi\epsilon_0 = 1$ where ϵ_0 is the permittivity of free space.

In the standard surface roughness picture, the liquid interacts with the solid through long-ranged van der Waals attraction and short-range Pauli repulsion, which we model with a 12-6 Lennard-Jones potential between the oxygen atoms on the water and the carbon atoms in the graphene sheet

$$\mathcal{U}_{\mathrm{SR}}(\boldsymbol{R}_{\mathrm{wat}}^N, \boldsymbol{R}_{\mathrm{sol}}^M) = \sum_{i}^{N} \sum_{j}^{M} u_{\mathrm{LJ}}(|\boldsymbol{r}_{\mathrm{O},i} - \boldsymbol{r}_{\mathrm{C},j}|), \tag{S3}$$

where $\boldsymbol{R}_{\mathrm{sol}}^M$ denotes the set of atomic positions for a configuration of M carbon atoms. Here, for simplicity, we fix the positions of the carbon atoms.

Polarization of the solid can then be incorporated with the classical Drude oscillator model.[1] Each carbon core now carries a positive charge $+Q_{\mathrm{D}}$ and is attached to a Drude particle of charge $-Q_{\mathrm{D}}$ and mass m_{D} through a harmonic spring with force constant k_{D}. The potential energy function of the solid then has the form

$$\mathcal{U}_{\mathrm{sol}}(\boldsymbol{R}_{\mathrm{sol}}^M) = \sum_{i}^{M} \frac{1}{2} k_{\mathrm{D}} |\boldsymbol{r}_{\mathrm{C},i} - \boldsymbol{r}_{\mathrm{D},i}|^2 + \sum_{i<j}^{M} \sum_{\alpha,\beta} \frac{Q_{\alpha,i} Q_{\beta,j}}{|\boldsymbol{r}_{\alpha,i} - \boldsymbol{r}_{\beta,j}|} \phi(|\boldsymbol{r}_{\alpha,i} - \boldsymbol{r}_{\beta,j}|). \tag{S4}$$

The first set of sums describes the total harmonic interaction of all Drude oscillators in the solid. The second set of sums describes the electrostatic interactions between the oscillators. To avoid the "polarization catastrophe", a Thole function,[2] $\phi(r)$, is used to damp Coulomb interactions at short distances. This function has the form

$$\phi(r) = 1 - \left(1 + \frac{sr}{2}\right) e^{-sr}, \tag{S5}$$

where the scaling coefficient s is determined by the polarizability of the carbon atom α_{C} and a Thole damping parameter δ_{C} via $s = \delta_{\mathrm{C}}/(\alpha_{\mathrm{C}})^{1/3}$.

In the absence of a field, each Drude particle oscillates around its core atom position $\boldsymbol{r}_{\mathrm{C}}$. In an electric field $\boldsymbol{E}(t)$, here arising from the fluctuating charge density of water, the Drude particle oscillates around $\boldsymbol{r}_{\mathrm{C}} + \boldsymbol{d}(t)$ where $\boldsymbol{d}(t)$ is the displacement of Drude particle from the core atom at time t and is given by $\boldsymbol{d}(t) = Q_{\mathrm{D}}\boldsymbol{E}(t)/k_{\mathrm{D}}$. The instantaneous induced dipole from the Drude oscillator is $\boldsymbol{\mu}_{\mathrm{D}}(t) = Q_{\mathrm{D}}\boldsymbol{d}(t) = Q_{\mathrm{D}}^2\boldsymbol{E}(t)/k_{\mathrm{D}}$. The isotropic atomic polarizability is then seen to be[1,3–5]

$$\alpha_{\mathrm{C}} = \frac{Q_{\mathrm{D}}^2}{k_{\mathrm{D}}}. \tag{S6}$$

The polarization response of the solid is therefore controlled by the parameters Q_{D} and k_{D}.

Introduction of point charges in the solid now introduces electrostatic interactions between the solid and the liquid, which we refer to as the charge density coupling term:

$$\mathcal{U}_{\mathrm{CC}}(\boldsymbol{R}_{\mathrm{wat}}^{N}, \boldsymbol{R}_{\mathrm{sol}}^{M}) = \sum_{i}^{N}\sum_{j}^{M}\sum_{\alpha,\beta} \frac{Q_{\alpha,i}Q_{\beta,j}}{|\boldsymbol{r}_{\alpha,i} - \boldsymbol{r}_{\beta,j}|}. \tag{S7}$$

The overall potential energy of the system is:

$$\mathcal{U}_{\mathrm{tot}}(\boldsymbol{R}_{\mathrm{wat}}^{N}, \boldsymbol{R}_{\mathrm{sol}}^{M}) = \mathcal{U}_{\mathrm{wat}}(\boldsymbol{R}_{\mathrm{wat}}^{N}) + \mathcal{U}_{\mathrm{SR}}(\boldsymbol{R}_{\mathrm{wat}}^{N}, \boldsymbol{R}_{\mathrm{sol}}^{M}) + \mathcal{U}_{\mathrm{sol}}(\boldsymbol{R}_{\mathrm{sol}}^{M}) + \mathcal{U}_{\mathrm{CC}}(\boldsymbol{R}_{\mathrm{wat}}^{N}, \boldsymbol{R}_{\mathrm{sol}}^{M}). \tag{S8}$$

We reiterate that in previous work,[6,7] classical treatments of liquid–solid interfacial friction have focused on the first two terms of the potential. Here, our model considers also interactions between fluctuating charge densities in the liquid and the solid through the addition of the last two terms.

S1.2. System set-up

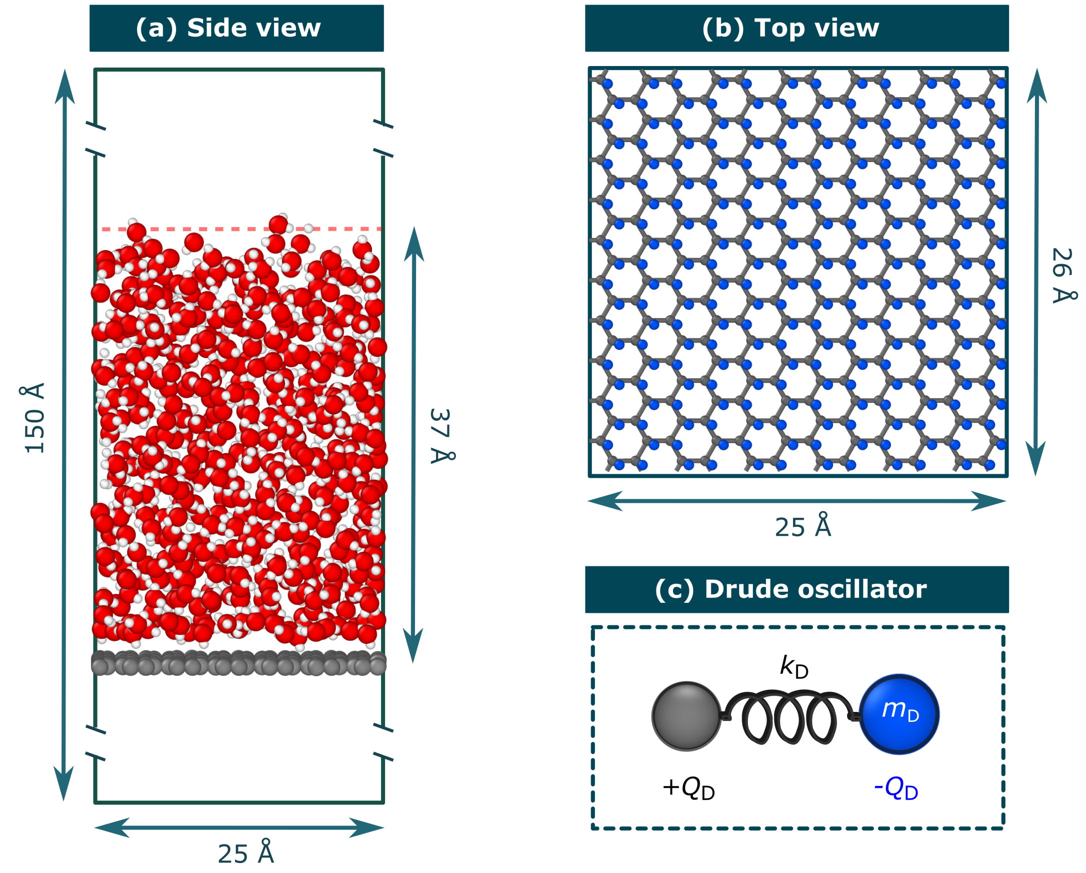

Figure S1. **System set-up.** The view from (a) the side of the liquid–solid interface and (b) the top of the solid sheet. Oxygen, hydrogen, carbon atoms and Drude particles are in red, white, grey and blue, respectively. The dark solid lines represent the edges of the simulation box. (c) The classical Drude oscillator is used to model charge density in the solid, whose parameters include: the harmonic spring constant k_{D}, the Drude mass m_{D}, charge on the core carbon atom $+Q_{\mathrm{D}}$ and on the Drude particle $-Q_{\mathrm{D}}$.

For the results presented in the main article, the simulations were carried out with 724 water molecules in a thin film of thickness ≈ 37 Å above a graphene layer of 240 carbon atoms, as illustrated in Fig. S1. Each carbon atom is attached to one Drude particle so there are 240 Drude particles. The orthorhombic cell has dimension $\approx 26 \times 25 \times 150$ Å^3. In addition, simulations of larger system sizes and of water films

with different thicknesses were also performed to check the sensitivity of the results, which are presented in Sec. S3.

S1.3. Simulation details

All simulations were carried out with the `LAMMPS` simulations package.[8,9] Water–water interactions were described with the SPC/E water model.[10] The geometry of water molecules was constrained using the `RATTLE` algorithm.[11] The carbon positions of the sheet were fixed. Water–carbon interaction responsible for the surface roughness was modeled with Werder parameters.[12] Charge densities on the graphene sheet were modeled using the classical Drude oscillator model[1,13] with Thole damping[2] using parameters from Misra and Blankschtein.[14] The value of parameters for all interaction potentials in the simulations are summarized in Tabs. S1 and S2. All Lennard-Jones interactions were truncated and shifted at 10 Å. Electrostatic interactions were cut off at 10 Å and long-ranged interactions were evaluated using particle–particle particle–mesh Ewald summation[15] such that the RMS error in the forces was a factor of 10^5 smaller than the force between two unit charges separated by a distance of 1.0 Å.[16] Drude mass choices to change the solid charge density frequency are in the range of $1 - 10^7$ amu.

Subsystem	Atom type	Mass [amu]	Charge [e]
water	O	15.9994	−0.8476
	H	1.008	+0.4238
carbon	C	*	+1.8520
	D	$1 - 10^7$	−1.8520

Table S1. **Parameters for masses and charges for atoms in simulations.** Parameters for water are from the SPC/E model[10] and those for the carbon solid are from Misra and Blankschtein.[14] The * denotes that C atoms are fixed so their masses do not contribute to the dynamics. The letter D is used to denote Drude particles.

Interaction potential	Potential form	Parameters
SPC/E[10]	rigid bond	$r_{\mathrm{OH}} = 1.0$ Å
	rigid angle	$\theta_{\mathrm{HOH}} = 109.47°$
	Lennard-Jones	$\epsilon_{\mathrm{OO}} = 0.1553\,\mathrm{kcal\,mol^{-1}}$
		$\sigma_{\mathrm{OO}} = 3.166$ Å
Werder[12]	Lennard-Jones	$\epsilon_{\mathrm{CO}} = 0.1553\,\mathrm{kcal\,mol^{-1}}$
		$\sigma_{\mathrm{CO}} = 3.190$ Å
Misra and Blankschtein[14]	Thole damping[2]	$\delta_{\mathrm{C}} = 1.507$
	harmonic bond	$k_{\mathrm{D}} = 1000\,\mathrm{kcal\,mol^{-1}\,Å^{-2}}$

Table S2. **Parameters for interaction potentials (force fields) employed in simulations.** For polarization in the solid, the choice of k_{D} and Q_{D} from Misra and Blankschtein[14] gives an isotropic polarizability of $\alpha_{\mathrm{C}} = 1.139\,\mathrm{Å}^3$.

The simulations were carried out in the canonical (NVT) ensemble, where the temperature was held at 300 K. Two separate Nosé–Hoover thermostats[17,18] were applied to the water and Drude particles. Each thermostat is a Nosé–Hoover chain with 10 thermostats and a damping constant of 0.1 ps. Dynamics were propagated using the velocity Verlet algorithm with a time-step of 1 fs, unless specified otherwise. Each system was equilibrated for 100 ps and the subsequent 10 ns was used for analysis to give the results presented in the main article. The sensitivity of the results to different simulation settings is presented in Sec. S3.

S2. COMPUTATION OF PROPERTIES

S2.1. Friction coefficient

For each equilibrium MD simulation, the friction coefficient was evaluated through the Green–Kubo formula[19] involving the time integral of the force autocorrelation function defined as:

$$\lambda_{\mathrm{GK}}(\tau) = \frac{1}{\mathcal{A}k_{\mathrm{B}}T}\int_0^\tau \mathrm{d}t\,\langle \mathcal{F}(0)\cdot\mathcal{F}(t)\rangle, \tag{S9}$$

where $\mathcal{A}$ is the interfacial lateral area, $\langle\cdots\rangle$ indicates an ensemble average and $\mathcal{F}(t)$ denotes the instantaneous lateral force exerted on the liquid by the solid at time t. $\mathcal{F}(t)$ is evaluated as the total summed force acting on all water molecules of a given configuration averaged over both in-plane dimensions (x, y) and is saved at every time-step (1 fs). In principle, the friction coefficient of the system is recovered at the long-time limit:

$$\lambda = \lim_{\tau\to\infty}\lambda_{\mathrm{GK}}(\tau). \tag{S10}$$

However, at long times, the integral in Eq. S9 decays to zero due to the finite lateral extent of the system[20,21] so evaluating $\lambda_{\mathrm{GK}}(\tau)$ to a plateau is commonly employed.[7,22,23] It has been shown that taking the maximum of $\lambda_{\mathrm{GK}}(\tau)$ only recovers friction correctly when there is a separation of timescales between the decay time and the memory time of the force autocorrelation function.[24] Since we are probing behaviors of the interface where there is no separation of timescales, we approximate λ as the plateaued friction coefficient averaged between correlation time of $5-10$ ps. Justification of this choice is detailed in Sec. S3. For each Drude mass, we perform equilibrium MD simulations to extract λ. The error bars correspond to the statistical errors obtained from splitting the entire trajectory into 100 blocks such that each block is 100 ps long.

In the main text, we decompose the static and dynamical components of the friction coefficient by reformulating the Green–Kubo expression in terms of the mean square force $\langle\mathcal{F}^2\rangle$ and the force decorrelation time τ_{F}:

$$\lambda = \frac{1}{\mathcal{A}k_{\mathrm{B}}T}\langle\mathcal{F}^2\rangle\tau_{\mathrm{F}}, \tag{S11}$$

where

$$\tau_{\mathrm{F}} = \int_0^\infty \mathrm{d}t\,\frac{\langle\mathcal{F}(0)\mathcal{F}(t)\rangle}{\langle\mathcal{F}^2\rangle}. \tag{S12}$$

In practice, we computed the friction coefficient and the mean square force first before obtaining the force decorrelation time via $\tau_{\mathrm{F}} = \lambda\mathcal{A}k_{\mathrm{B}}T/\langle\mathcal{F}^2\rangle$.

S2.2. Surface response function

In the main article, the charge density distributions of the solid and the liquid are characterized by their surface response functions defined as

$$\operatorname{Im} g_{\mathrm{sol}}(q,\omega) = \frac{\pi\omega}{q\mathcal{A}k_{\mathrm{B}}T}\int_{-\infty}^{\infty}\mathrm{d}t\,\mathrm{e}^{i\omega t}\sum_{\alpha,\beta\,\in\,\mathrm{sol}}\Big\langle Q_\alpha Q_\beta\,\mathrm{e}^{-i\boldsymbol{q}\cdot[\boldsymbol{x}_\alpha(t)-\boldsymbol{x}_\beta(0)]}\mathrm{e}^{-q|z_\alpha(t)-z_0|}\,\mathrm{e}^{-q|z_\beta(0)-z_0|}\Big\rangle, \tag{S13}$$

and

$$\operatorname{Im} g_{\mathrm{wat}}(q,\omega) = \frac{\pi\omega}{q\mathcal{A}k_{\mathrm{B}}T}\int_{-\infty}^{\infty}\mathrm{d}t\,\mathrm{e}^{i\omega t}\sum_{\alpha,\beta\,\in\,\mathrm{wat}}\Big\langle Q_\alpha Q_\beta\,\mathrm{e}^{-i\boldsymbol{q}\cdot[\boldsymbol{x}_\alpha(t)-\boldsymbol{x}_\beta(0)]}\mathrm{e}^{-q|z_\alpha(t)-z_0|}\,\mathrm{e}^{-q|z_\beta(0)-z_0|}\Big\rangle, \tag{S14}$$

respectively. Here, Q_α is the charge on atom α, whose position in the plane of the graphene sheet at time t is $\boldsymbol{x}_\alpha(t)$, $\boldsymbol{q}$ is a wavevector parallel to the graphene sheet, $z_\alpha(t)$ is the vertical coordinate and $z_0 = 1.6$ Å above the graphene sheet defines a plane between carbon atoms and the water contact layer.

In practice, we computed at every time-step (1 fs) the Fourier–Laplace surface components of the charge densities for the solid and the liquid, defined as

$$\tilde{n}^{(\mathrm{s})}_{\mathrm{sol}}(q,t) = \sum_{\alpha\,\in\,\mathrm{sol}} Q_\alpha \mathrm{e}^{i\boldsymbol{q}\cdot\boldsymbol{x}_\alpha(t)} \mathrm{e}^{-q|z_\alpha(t)-z_0|}, \tag{S15}$$

and

$$\tilde{n}^{(\mathrm{s})}_{\mathrm{wat}}(q,t) = \sum_{\alpha\,\in\,\mathrm{wat}} Q_\alpha \mathrm{e}^{i\boldsymbol{q}\cdot\boldsymbol{x}_\alpha(t)} \mathrm{e}^{-q|z_\alpha(t)-z_0|}, \tag{S16}$$

respectively. As we are interested in the long-wavelength limit ($q \to 0$), we focus on $\boldsymbol{q} = \boldsymbol{q}_0$, the lowest wavevector in the x direction accessible in our simulation box, the magnitude of which is $q_0 = 2\pi/L_x \approx 0.25\,\text{Å}^{-1}$ where L_x is the length of the box in the x direction. The power spectra of the surface charge densities are given as

$$S^{(\mathrm{s})}_{\mathrm{sol}}(q,\omega) = \frac{1}{\mathcal{A}} \int_{-\infty}^{+\infty} \mathrm{d}t \, \langle \tilde{n}^{(\mathrm{s})}_{\mathrm{sol}}(q,0)\, \tilde{n}^{(\mathrm{s})}_{\mathrm{sol}}(-q,t) \rangle \, \mathrm{e}^{i\omega t}, \tag{S17}$$

and

$$S^{(\mathrm{s})}_{\mathrm{wat}}(q,\omega) = \frac{1}{\mathcal{A}} \int_{-\infty}^{+\infty} \mathrm{d}t \, \langle \tilde{n}^{(\mathrm{s})}_{\mathrm{wat}}(q,0)\, \tilde{n}^{(\mathrm{s})}_{\mathrm{wat}}(-q,t) \rangle \, \mathrm{e}^{i\omega t}. \tag{S18}$$

Through the fluctuation-dissipation theorem, we can obtain the imaginary part of the surface response function through:

$$\mathrm{Im}\, g_{\mathrm{sol}}(q,\omega) = \frac{2\pi}{q} \frac{\omega}{2k_{\mathrm{B}}T} S^{(\mathrm{s})}_{\mathrm{sol}}(q,\omega), \tag{S19}$$

and

$$\mathrm{Im}\, g_{\mathrm{wat}}(q,\omega) = \frac{2\pi}{q} \frac{\omega}{2k_{\mathrm{B}}T} S^{(\mathrm{s})}_{\mathrm{wat}}(q,\omega). \tag{S20}$$

To ensure the spectrum is independent of noise, a Savitzky–Golay filter[25] was applied. The resulting spectra without further fitting are presented in the main article.

S2.3. Surface charge density autocorrelation function

To characterize relaxation of the solid and the liquid charge densities, we computed their respective normalized autocorrelation functions, defined as:

$$C^{(\mathrm{s})}_{\mathrm{sol}}(\tau;q) = \frac{\langle \tilde{n}^{(\mathrm{s})}_{\mathrm{sol}}(q,0)\, \tilde{n}^{(\mathrm{s})}_{\mathrm{sol}}(-q,\tau) \rangle}{\langle |\tilde{n}^{(\mathrm{s})}_{\mathrm{sol}}(q)|^2 \rangle}, \tag{S21}$$

and

$$C^{(\mathrm{s})}_{\mathrm{wat}}(\tau;q) = \frac{\langle \tilde{n}^{(\mathrm{s})}_{\mathrm{wat}}(q,0)\, \tilde{n}^{(\mathrm{s})}_{\mathrm{wat}}(-q,\tau) \rangle}{\langle |\tilde{n}^{(\mathrm{s})}_{\mathrm{wat}}(q)|^2 \rangle}. \tag{S22}$$

Again, focusing on the long-wavelength limit, we show the results for $C^{(\mathrm{s})}_{\mathrm{sol}}(\tau;q_0)$ and $C^{(\mathrm{s})}_{\mathrm{wat}}(\tau;q_0)$ in the main article.

S3. SENSITIVITY OF THE FRICTION COEFFICIENT

To assess the robustness of our results to the choice of simulation settings, here we present an extensive set of tests on the sensitivity of the friction coefficients computed to certain aspects of our simulations. In these tests, we show the results for two representative cases: $m_{\mathrm{D}} = 1\,\mathrm{amu}$ for the weak-coupling regime and $m_{\mathrm{D}} = 5000\,\mathrm{amu}$ for the strong-coupling regime.

S3.1. Convergence of the Green–Kubo friction coefficient

As seen from Fig. S2, the force autocorrelation $\langle \mathcal{F}(0)\mathcal{F}(\tau)\rangle$ shows oscillations due to charge density fluctuations in the solid at short timescales before decaying to zero at longer timescales. We can characterize two particular timescales: (i) the time at which the first minimum is reached, $\tau_{\mathrm{D}} \propto \omega_{\mathrm{D}}$, due to motion of the Drude oscillators and (ii) the force decorrelation time, $\tau_{\mathrm{F}} \propto \lambda$, as defined by Eq. S12. These are marked for the weak-coupling case in the inset of Fig. S2(a). In $\lambda_{\mathrm{GK}}(\tau)$, these timescales manifest in a peak at τ_{D} and a plateau after τ_{F}. In the weak-coupling regime where the Drude mass is low, there is a separation of timescales as $\tau_{\mathrm{D}} \ll \tau_{\mathrm{F}}$. A decrease in the solid charge density frequency in this regime only increases τ_{D} but not τ_{F}. The peak at τ_{D} is a local maximum, increasing in height as Drude mass increases, and a plateau is observed at times intermediate between τ_{D} and τ_{F}, unchanged in height. This is consistent with the converged friction coefficient remaining at $\lambda \approx 1.9 \times 10^4\,\mathrm{N\,s\,m^{-3}}$. In the strong-coupling regime where the solid frequency is slow, τ_{D} is now comparable to τ_{F}. A decrease in the solid charge density frequency in this regime increases both τ_{D} and τ_{F}. As τ_{F} governs the value of λ_{GK} at long times, the converged friction coefficient now increases with decreasing charge density frequency. In order to get a consistent value for the friction coefficient, it is important to extract λ from $\lambda_{\mathrm{GK}}(\tau)$ at $\tau > \tau_{\mathrm{F}}$. Therefore, taking the maximum of $\lambda_{\mathrm{GK}}(\tau)$ as in some studies[23,26,27] is not appropriate, as also shown previously by Oga *et al.*[24]

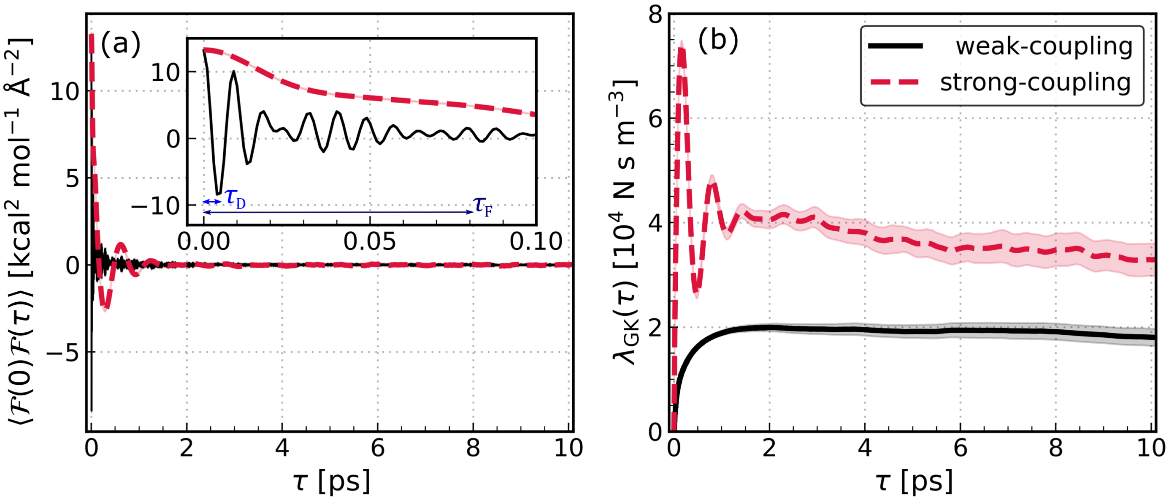

Figure S2. **Convergence of the Green–Kubo friction coefficient** (a) The force autocorrelation function of two representative cases in the weak-coupling and strong-coupling regimes. The inset shows clearer the oscillations due to the Drude dynamics. The memory time τ_{D} and the decay time τ_{F} are marked for the weak-coupling case. (b) The convergence of λ_{GK} where statistical errors (shaded area) are obtained from block-averaging.

For simulations with the largest Drude mass $m_{\mathrm{D}} = 10^7\,\mathrm{amu}$, we observe a plateau in $\lambda_{\mathrm{GK}}(\tau)$ from $\tau \gtrsim 5\,\mathrm{ps}$. Therefore, we give the converged friction coefficient in all cases to be the average of values of $\lambda_{\mathrm{GK}}(\tau)$ evaluated at correlation time between $5 < \tau/\,\mathrm{ps} < 10$.

S3.2. System size

To make sure the employed system size is sufficient to obtained converged values, we checked if our results remain consistent at larger system sizes. In Fig. S3, the friction coefficient for two representative Drude masses are shown for systems with different lateral areas in the (x, y) plane. The additional simulations were carried out on an interface with approximately the same liquid film thickness ≈ 37 Å. The friction coefficients computed from these simulations are almost identical to those presented in the main article.

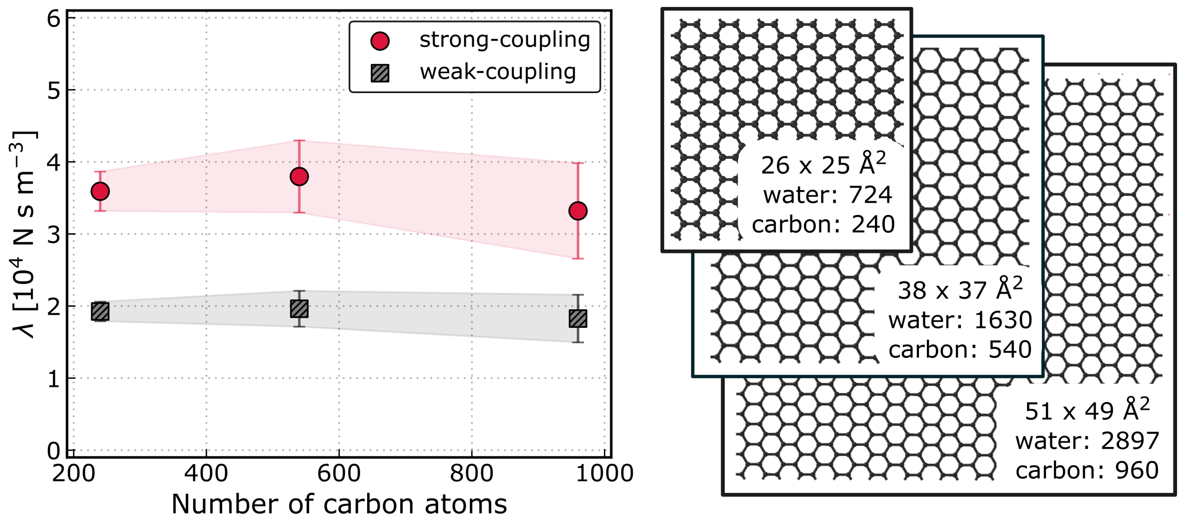

Figure S3. **Sensitivity of friction coefficient to system size.** Statistical errors are obtained from block-averaging. The three system sizes tested are illustrated on the right panel where only the solid sheets are shown. The corresponding total area for the sheet and the number of water molecules and carbon atoms are given for each system

S3.3. Simulation time

Analogous to checking the impact of system size, we checked the convergence of the friction coefficient with the simulation time length, as presented in Fig. S4. For all cases, the friction coefficient is found to change relatively little with increasing simulation time. While statistical errors are larger for shorter simulations, simulation times as short as 1 ns is enough to converge friction to within 10%. Therefore, the employed simulation time of 10 ns is sufficient for a converged friction coefficient.

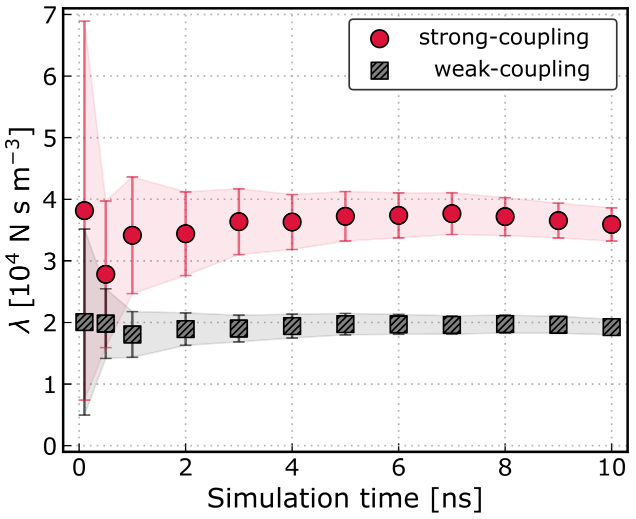

Figure S4. **Sensitivity of friction coefficient to simulation time.** λ changes relatively little with increasing simulation time. Statistical errors are obtained from block-averaging.

S3.4. Time-step

We test the sensitivity of the friction coefficients in both the weak-coupling and strong-coupling regimes with the time-step used in simulations and see that the results agree well for time-step of $0.1, 0.2, 0.5$ and 1 fs, as shown in Fig. S5(a). However, when dealing with small Drude masses, problems with energy drifts often arise due to the inherently high frequency of the individual Drude oscillators.[1] We therefore also check that the time-step of 1 fs employed gives acceptable value of λ for Drude masses of $0.4 \leq m_{\mathrm{D}}/\mathrm{amu} \leq 5$ in our simulations, as shown in Fig. S5(b). Here, all these simulations belong to the weak-coupling regimes.

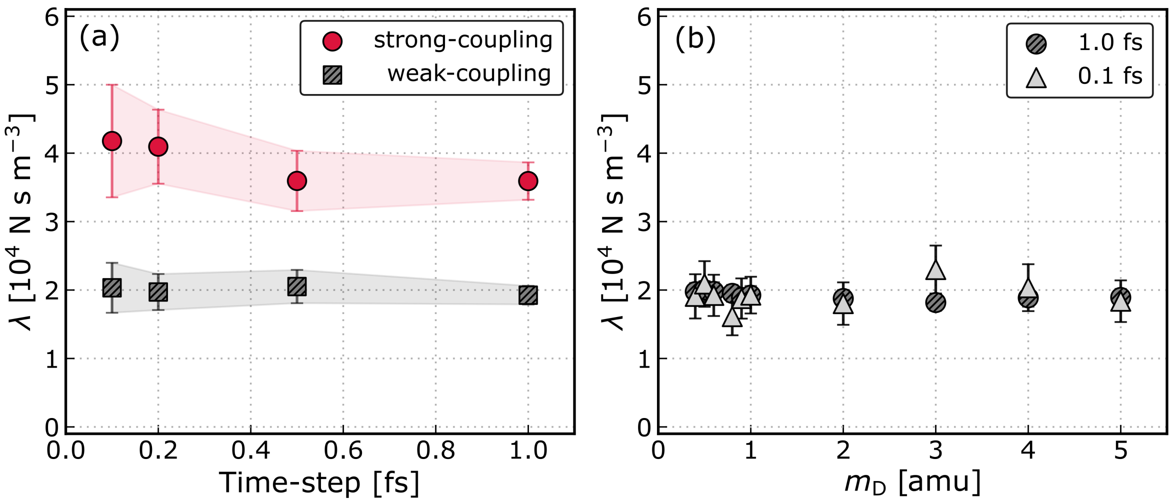

Figure S5. **Sensitivity of friction coefficient to time-step.** (a) Dependence of the computed λ on the time-step used in simulations. (b) Comparison of λ for simulations with $m_{\mathrm{D}} \lesssim 5$ amu using a time-step of 0.1 and 1 fs. Statistical errors are obtained from block-averaging.

Further analysis by extracting the static component, quantified by $\langle \mathcal{F}^2 \rangle$, and the dynamical component, quantified by τ_{F}, of the friction is shown in Fig. S6. This reveals that although λ converge for $m_{\mathrm{D}} \lesssim 2$ amu for a time-step of 1 fs, $\langle \mathcal{F}^2 \rangle$ and τ_{F} diverge for these small masses. Therefore, in the main article, $\langle \mathcal{F}^2 \rangle$ and τ_{F} for $m_{\mathrm{D}} \lesssim 2$ amu cases are computed from simulations using a time-step of 0.1 fs.

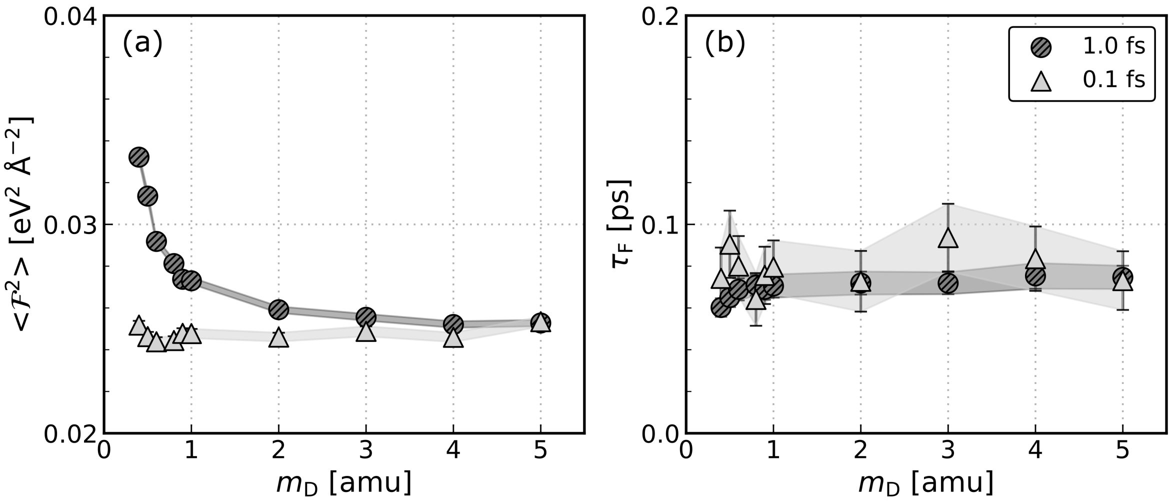

Figure S6. **Sensitivity of the static and dynamical components of friction to time-step.** Variation of (a) the mean squared force $\langle \mathcal{F}^2 \rangle$ and (b) the force decorrelation time τ_F for simulations with Drude masses $0.4 \leq m_D/\text{amu} \leq 5$ using a time-step of 0.1 and 1 fs.

S3.5. Thermostats

Ordinarily, in simulations of polarizable systems using the classical Drude oscillator model, the temperature of the Drude particles is often kept low[1,3,28] to minimize energy exchange between the nuclear and Drude motion. In this work, we found numerical instabilities in simulations with low T_{sol} for $m_D \gtrsim 10$ amu. Therefore, for all results presented in this work, we used two separate thermostats to keep the temperatures of the water, T_{wat}, and the Drude particles, T_{sol}, both at 300 K. We carried out simulations with $m_D = 1$ amu (the weak-coupling regime) using different values for $T_{sol} = 10, 100$ and 300 K. As shown in Fig. S7, while the force autocorrelation function shows oscillations of greater magnitude at higher T_{sol}, its integral and therefore the friction coefficient is not significantly affected.

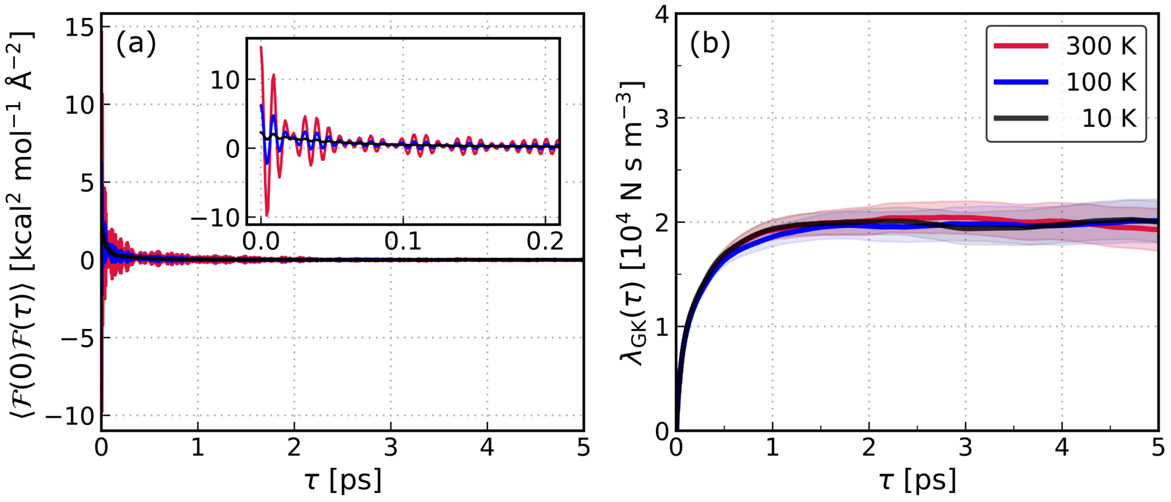

Figure S7. **Senstivity of friction coefficient to the temperature of the Drude particles.** (a) The force autocorrelation function for simulations with $m_D = 1$ amu where the Drude particles are kept at $T_{sol} = 10, 100, 300$ K. The inset shows clearer the oscillations at short times. (b) The convergence of λ_{GK} for the three cases where statistical errors (shaded area) are obtained from block-averaging.

We also test the sensitivity of the friction coefficient to the thermostat settings used to maintain the temperature of the Drude particles. In Fig. S8, we show that the friction coefficients agree well for

different damping times of the Nosé–Hoover thermostat for the Drude particles $\tau_{\mathrm{NH}} = 1, 10, 100, 1000$ fs in both the weak-coupling and strong-coupling regimes. Equivalent results were also obtained with the canonical sampling through velocity rescaling (CSVR) thermostat.[29]

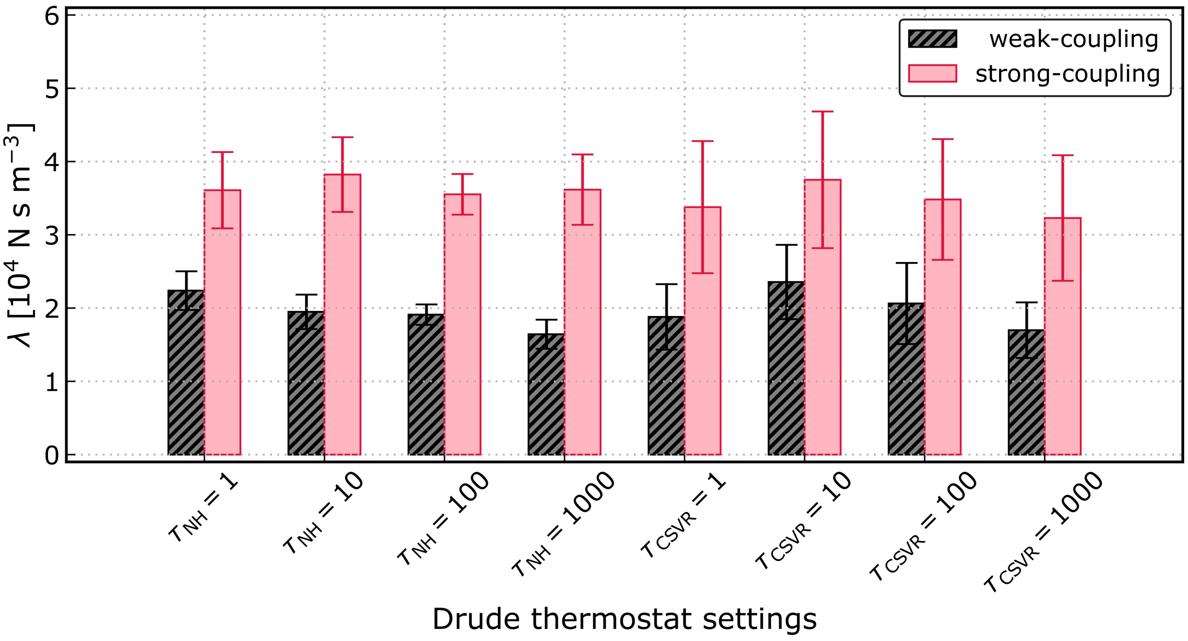

Figure S8. **Sensitivity of friction coefficient to thermostat settings for Drude particles.** The values of the damping time constant of the thermostat (τ_{NH} for the Nosé–Hoover thermostat and τ_{CSVR} for the CSVR thermostat) for the Drude particles are given in fs. Statistical errors are obtained from block-averaging.

S3.6. Liquid film thickness

Simulations for systems with varying thickness of the water film were performed to test the convergence of the friction coefficient. In Fig. S9(a), we show the planar average mass density profiles for the liquid–solid system with a water film of thickness $\approx 7, 17, 27, 37$ and 47 Å. The thickness is determined by the height from the positions of the carbon atoms in the sheet to where the water density at the liquid–vapour interface is equal to $0.5\,\mathrm{g\,cm^{-3}}$. In Fig. S9(b), we show how the extracted friction coefficient changes with the water film thickness. A water film with thickness $\gtrsim 17$ Å is required to have a region with bulk mass density and a converged friction coefficient. Therefore, employing a thickness of ≈ 37 Å for simulations of our main results is justified.

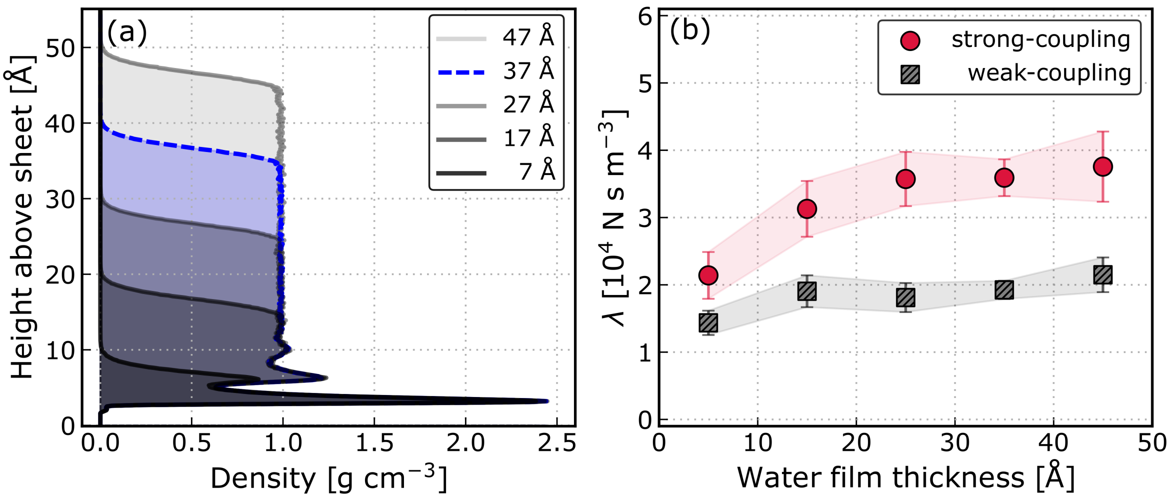

Figure S9. **Sensitivity of friction to simulation time to the liquid film thickness.** (a) The planar density profile of different film thicknesses above the flat solid sheet is shown for different water film thicknesses (indicated in the legend). In the main article, we use a thickness of ≈ 37 Å, as indicated by the dashed blue line. (b) The sensitivity of friction to the liquid thickness. Statistical errors are obtained from block-averaging.

S3.7. Electrostatic boundary conditions

For simulations presented in the main article, we treated electrostatics by applying the conventional three-dimensional Ewald summation (EW3D) technique.[30] This method is commonly employed in simulations of different interfacial systems.[23,31,32] To test the influence of this choice to the friction coefficient, we performed additional simulations with hybrid boundary conditions in which the Ewald summation is applied to the x and y directions while the electric displacement field in the z direction is set to zero ($D_z = 0$). This is done using the finite field approach[33–35] and such hybrid boundary conditions have been shown[33] to be formally equivalent to the Yeh–Berkowitz correction[36] that decouples the electrostatic interactions between a slab of material and its periodic images. We found good agreement for the friction coefficients of both the weak-coupling and strong-coupling regimes between the EW3D and the $D_z = 0$ methods, as shown in Fig. S10.

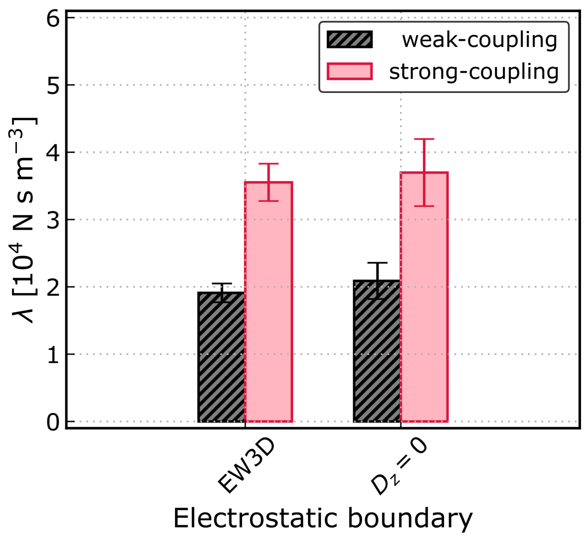

Figure S10. **Sensitivity of friction coefficient to electrostatic boundary conditions.** The friction coefficients in the weak-coupling and strong-coupling agree well for simulations employing the EW3D method and the $D_z = 0$ method. Statistical errors are obtained from block-averaging.

S4. SENSITIVITY OF THE DEPENDENCE OF FRICTION ON SOLID CHARGE DENSITY FREQUENCY

For the simulations presented in the main article, we fixed $Q_\mathrm{D} = 1.852\,e$ and $k_\mathrm{D} = 1000\,\mathrm{kcal\,mol^{-1}\,Å^{-2}}$ for the Drude oscillators, which have been parameterized by Misra and Blanckstein to recover the polarizability tensor of a periodic graphene lattice.[14] In principle, Q_D and k_D both control the solid charge density and changing their values will affect the coupling between the solid and the liquid charge densities, and therefore the friction. Here we will present a check for the sensitivity of the dependence of friction on solid charge frequency upon changing to different values for each of these parameters.

S4.1. Varying the Drude charge

We performed two additional sets of simulations of the liquid–solid interface with $Q_\mathrm{D} = 0$ and $0.926\,e$ while keeping $k_\mathrm{D} = 1000\,\mathrm{kcal\,mol^{-1}\,Å^{-2}}$ for Drude masses in the range $1 \lesssim m_\mathrm{D}/\mathrm{amu} \lesssim 10^7$ while other aspects of the simulations are kept the same. In the main article, we show the dependence of the friction on the frequency of the solid charge density by plotting λ against ω_0. Based on the discussion in Sec. S5, we can approximate the solid charge density as $\omega_0 \approx \omega_\mathrm{D}$ instead. Therefore, for convenience, in these additional analyses, λ is plotted against ω_D as shown in Fig. S11. The relationship mapped out for $Q_\mathrm{D} = 1.852\,e$ is essentially unchanged compared to the one presented in Fig. 2(a) in the main article.

For $Q_\mathrm{D} = 0$, there is no charge density in the solid, i.e $\tilde{n}_\mathrm{sol}(q,\omega) = 0$, so the only contribution to friction is from the surface roughness. Therefore the friction remains constant with ω_D at $\lambda \approx 1.7 \times 10^4\,\mathrm{N\,s\,m^{-3}}$ Increasing Q_D to 0.926 and $1.852\,e$ slightly increases the surface roughness contribution to friction, as seen from the flattening in the weak-coupling regime. Meanwhile, the contribution to friction from charge density coupling increases much more significantly in the strong-coupling regime for higher values of Q_D. This behavior supports the fact that the increase in friction at the low frequency end is indeed due to coupling of charge density between the solid and the liquid and larger charge density in the solid will couple more strongly with the liquid, resulting a larger increase in friction the strong-coupling regime.

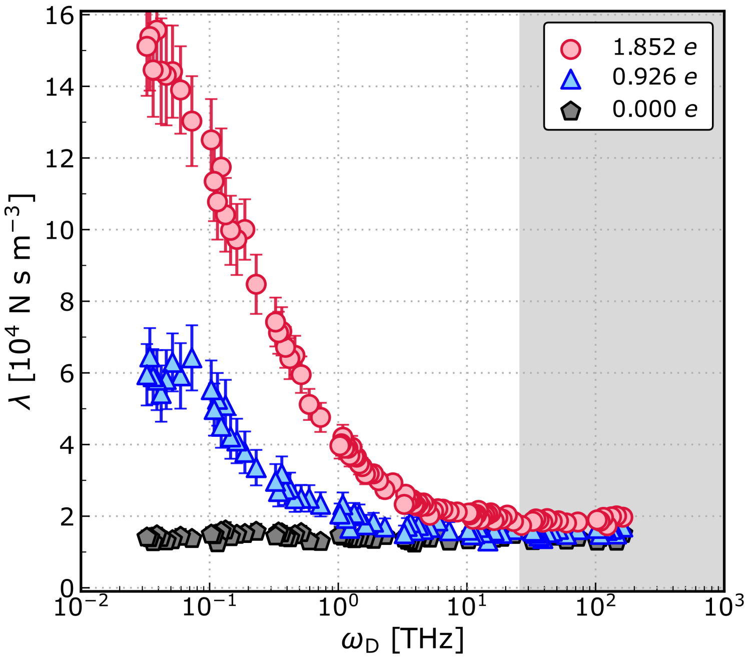

Figure S11. **Dependence of friction on solid charge density frequency for different Drude charges.** Values of Q_D are indicated in the legend and $k_\mathrm{D} = 1000\,\mathrm{kcal\,mol^{-1}\,Å^{-2}}$ in all cases. The weak-coupling and strong-coupling regimes are shaded grey and not shaded, respectively. For simulations with $Q_\mathrm{D} = 0$, λ remains constant with ω_D. For simulations with $Q_\mathrm{D} = 0.926$ and $1.852\,e$, λ increases as ω_D is decreased in the strong-coupling regime.

S4.2. Varying the spring constant

Here, we performed two additional sets of simulations of the liquid–solid interface with $k_\mathrm{D} = 600$ and $1000\,\mathrm{kcal\,mol^{-1}\,Å^{-2}}$ while keeping $Q_\mathrm{D} = 1.852\,e$, also for Drude masses in the range $1 \lesssim m_\mathrm{D}/\mathrm{amu} \lesssim 10^7$, while other aspects of the simulations are kept the same.

Again, since the solid charge density is changed when k_D is varied, differences in the absolute values of λ are expected and indeed observed, as shown in Fig. S12. According to Eq. S6, a decrease in k_D means that the atom modeled by the Drude oscillator becomes less polarizable. Therefore, the friction contribution due to the surface roughness is higher for lower values of k_D but the differences in the λ in the weak-coupling regime are relatively small. The contribution to friction from charge density coupling is observed to be more strongly affected since λ increases much more sharply for lower k_D as ω_D is decreased in the strong-coupling regime. The important thing to stress is that this increase of λ due to charge density coupling in all cases occur once $\omega_\mathrm{D} \lesssim \omega_\mathrm{lib}$, supporting the separation into the weak-coupling and strong-coupling regimes in the main article.

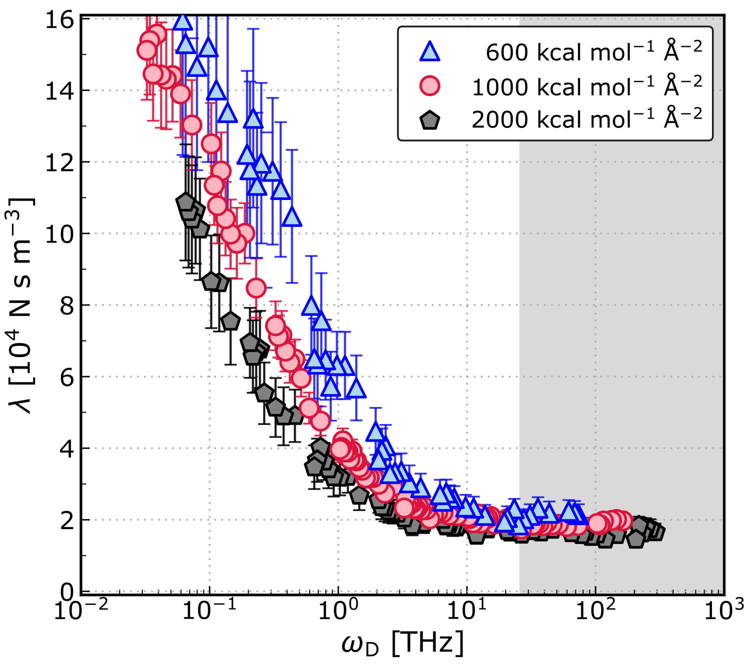

Figure S12. **Dependence of friction on solid charge density frequency for different harmonic force constant.** Values of k_D are indicated in the legend and $Q_\mathrm{D} = 1.852\,e$ in all cases. The weak-coupling and strong-coupling regimes are shaded grey and not shaded, respectively. For all cases, λ increases once $\omega_\mathrm{D} \lesssim \omega_\mathrm{lib}$ in the strong-coupling regime.

S4.3. Flexible water model

To check the sensitivity of our results to the presence of intramolecular modes in water, we performed additional simulations with a flexible water model SPC/Fw.[37] In additional to the intermolecular librational and Debye modes, SPC/Fw also captures the OH stretching modes as a peak centered at $\approx 100\,\mathrm{THz}$ and in-plane bending modes as a peak centered at $\approx 50\,\mathrm{THz}$. The addition of these peaks in the water dielectric spectrum does not affect the conclusions drawn in the main article, as shown in Fig. S13.

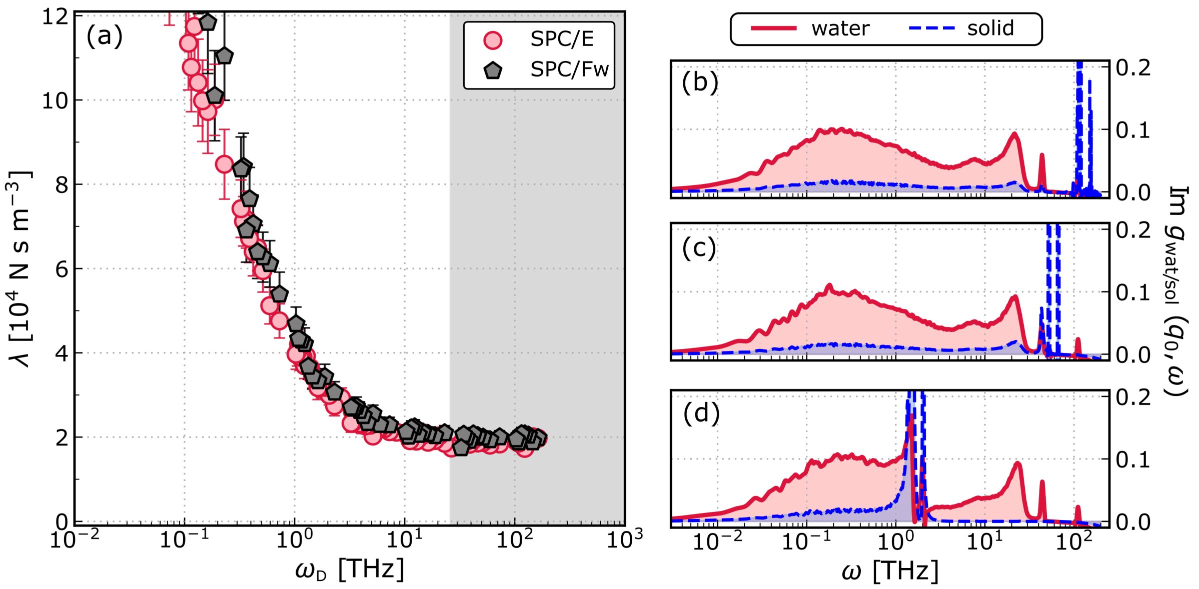

Figure S13. **Dependence of friction on solid charge density frequency for a flexible water model.** (a) The friction dependence on ω_{D} remains almost unchanged when simulations are performed with a rigid (SPC/E) or flexible (SPC/Fw) water model. When solid principal peaks coincide with (b) the OH stretching modes or (c) the in-plane stretching mode, there is no significant response in either the solid or the liquid. (d) The strong-coupling regime remains at frequencies lower than the librational peak.

S4.4. Phonon contribution

In this work, we do not consider the effect of phonons in the solid on the friction of the interface, which would in principle affect both the surface roughness contribution λ_{SR} and the contribution from the coupling of the dynamics of the solid and the liquid λ_{THz}. Here, we can simply investigate how a single phonon mode in the solid would change the friction. We do this by considering a simple model in which the solid is a set of independent harmonic oscillators whereby the carbon atoms are now attached to their lattice positions via a harmonic spring with force constant k_{ph}, and ascribed a mass m_{ph}. The carbon atoms still interact with the water through the same Lennard-Jones potential. By varying m_{ph} we can tune the phonon frequency $\omega_{\mathrm{ph}} = (k_{\mathrm{ph}}/m_{\mathrm{ph}})^{1/2}$ (with $k_{\mathrm{ph}} = 1000\,\mathrm{kcal\,mol^{-1}\,\mathring{A}^{-2}}$) in a similar fashion to how we tuned the solid's dielectric spectrum. In contrast to varying m_{D}, we see that friction is relatively insensitive to changes in m_{ph}, as seen in Fig. S14. This result is in line with the prediction of QF theory that, for water at carbon substrates, phonon contributions to quantum friction are relatively small.

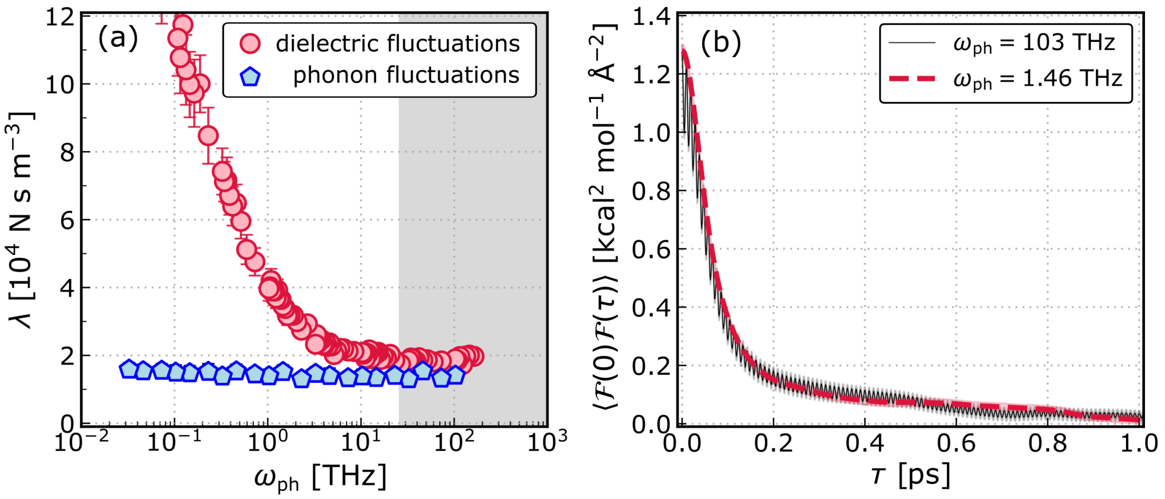

Figure S14. **Dependence of the friction on the phonon mode frequency.** (a) The friction is not strongly affected by phonon fluctuations compared to the effect seen for dielectric fluctuations in the the solid. (b) The force–force autocorrelation functions are shown for two different phonon frequencies as indicated in the legend. Unlike for dielectric modes, the phonon modes do not change the force decorrelation time significantly.

S5. COUPLING OF CHARGE DENSITIES

In this section, we present further analyses of the surface response functions of the solid and the liquid in support of the conclusions made on the separation into the weak-coupling and strong-coupling regimes in the main article.

S5.1. Solid surface response function in the absence of water

In the main article, we show that the surface response function of the solid $g_{\mathrm{sol}}(q_0, \omega)$ is dominated by two peaks that are slightly blue-shifted from ω_{D}. Here we provide more details into the origin of each of these peaks, with the peak lower in frequency denoted as ω_0 and the peak higher in frequency denoted as ω_1.

In the absence of the Coulomb interaction, the Drude oscillators are simply a set of independent harmonic oscillators characterized by a single frequency ω_{D}. In the presence of the Coulomb interaction, the motion of the Drude oscillators is no longer isotropic, with motion perpendicular to the plane of the graphene sheets having a higher frequency (ω_1) than parallel motion (ω_0). To illustrate this point further, in Fig. S15(b), we show how $g_{\mathrm{sol}}(q_0, \omega)$ changes as the force constants of the springs are changed to $(k_{xy}, k_z) = (4k_{\mathrm{D}}, k_{\mathrm{D}})$ and $(k_{xy}, k_z) = (k_{\mathrm{D}}, 4k_{\mathrm{D}})$, where $k_{\mathrm{D}} = 1000\,\mathrm{kcal\,mol^{-1}\,\AA^{-2}}$. (To be clear, in the main article, an isotropic spring constant k_{D} is used throughout.) When $(k_{xy}, k_z) = (4k_{\mathrm{D}}, k_{\mathrm{D}})$, ω_0 shifts to higher frequency and ω_1 is unaffected. Conversely, when $(k_{xy}, k_z) = (k_{\mathrm{D}}, 4k_{\mathrm{D}})$, ω_1 shifts to higher frequency and ω_0 position is unaffected.

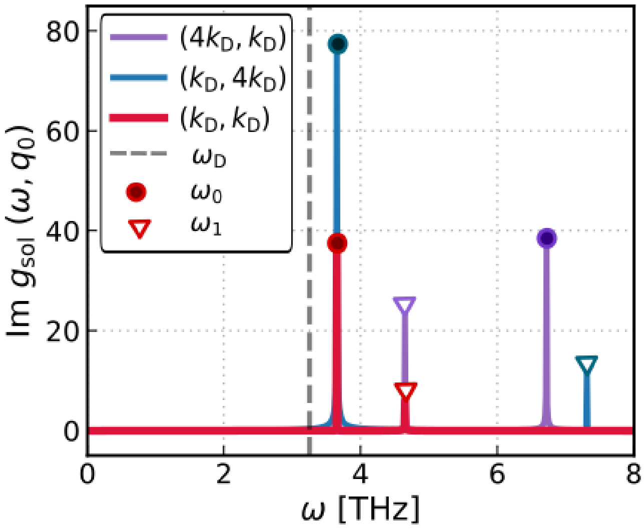

Figure S15. **Solid surface charge density in the absence of water.** The surface response function $g_{\mathrm{sol}}(q_0, \omega)$ of a carbon sheet with Drude particles of mass $m_{\mathrm{D}} = 10^3$ amu such that $\omega_{\mathrm{D}} = (k_{\mathrm{D}}/m_{\mathrm{D}})^{1/2} = 3.3\,\mathrm{THz}$ in the absence of water. The value of ω_{D} is marked with a dashed vertical line. When the spring is changed from having an isotropic force constant $(k_{xy}, k_z) = (k_{\mathrm{D}}, k_{\mathrm{D}})$ to an anisotropic one, the modes in $g_{\mathrm{sol}}(q_0, \omega)$ are shifted accordingly. When $(k_{xy}, k_z) = (4k_{\mathrm{D}}, k_{\mathrm{D}})$, the parallel mode at ω_0 (denoted with a filled circle) is shifted. When $(k_{xy}, k_z) = (k_{\mathrm{D}}, 4k_{\mathrm{D}})$, the perpendicular mode at ω_1 (denoted with an empty triangle) is shifted.

To access the dispersion relation of the solid modes in more detail, we perform simulations of the solid system with a supercell length of 153.36 Å. In Fig. S16(a), we show $g_{\mathrm{sol}}(q, \omega)$ in both q-space and ω-space. Both the solid modes show relatively flat dispersion, with very narrow widths in ω-space and spanning up to $q_{\mathrm{max}} \approx 1.5\mathrm{\AA^{-1}}$ in q-space. This flat dispersion of the solid modes is seen in simulations with different values of ω_{D}, as illustrated in Fig. S16(b).

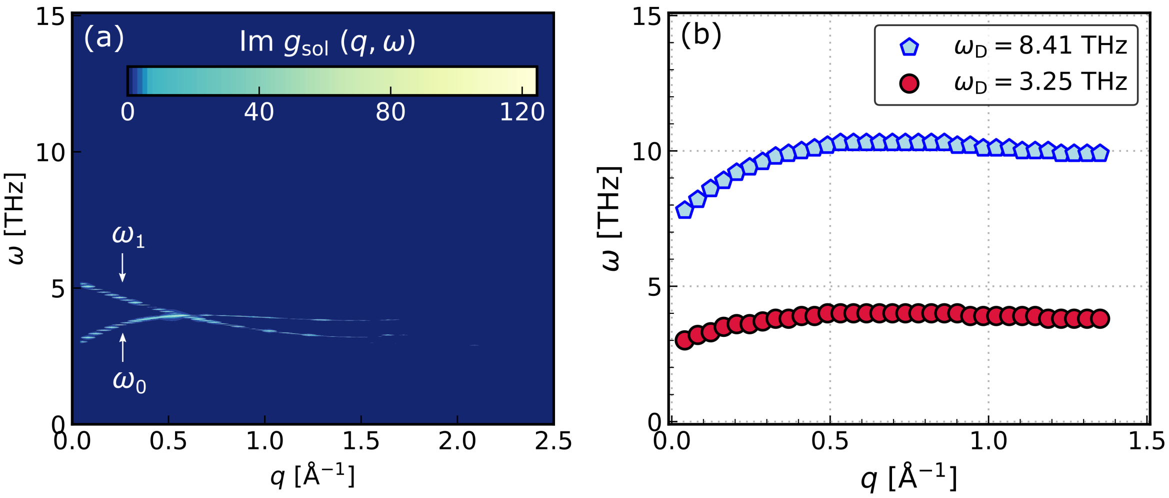

Figure S16. **Dispersion relation of the solid modes.** (a) The solid surface response $g_{\mathrm{sol}}(q, \omega)$ of a carbon sheet with Drude particles of mass $m_{\mathrm{D}} = 10^3$ amu in the absence of water. The tangential mode increases in frequency while the perpendicular mode decreases in frequency before flattening out up to $q_{\max} \approx 1.5\text{Å}^{-1}$. (b) The solid tangential mode shows relatively flat dispersion relation, as shown for two representative simulations with ω_{D} indicated in the legend.

S5.2. Solid surface response function in the presence of water

In the absence of water, there are two sources of dissipation in the solid as the Drude oscillators are coupled to a thermostat and also interact with each other by electrostatic interactions. Therefore, the principal modes in the solid have finite widths in the response function. However, since the Drude oscillators are only weakly-coupled, they remain underdamped and these widths remain very small. In the presence of water, when there is strong-coupling with the intermolecular modes of water, the Drude oscillators are more strongly damped, leading to the broadening of their widths.

Focusing on the case when $\omega_{\mathrm{D}} = 3.26\,\mathrm{THz}$, we can quantify this broadening by fitting the surface response function $g_{\mathrm{sol}}(q_0, \omega)$ obtained from simulations to a double-Lorentzian

$$\mathrm{Im}\, g_{\mathrm{sol}}(q_0, \omega) = \frac{a_0 \eta_0^2}{(\omega - \omega_0)^2 + \eta_0^2} + \frac{a_1 \eta_1^2}{(\omega - \omega_1)^2 + \eta_1^2}, \tag{S23}$$

where a_0 is the amplitude, ω_0 is the centered frequency, η_0 is the width of the tangential mode and a_1, ω_1, η_1 are similarly defined for the perpendicular mode. The results obtained for the fitting parameter are given in Table S3. We see that both of the principal modes experience a small redshift to lower frequency of $\approx 0.06\,\mathrm{THz}$. More prominently, the amplitudes of both peaks are reduced and the widths are broadened in the presence of water.

	a_0	ω_0 [THz]	η_0 [THz]	a_1	ω_1 [THz]	η_1 [THz]
without water	17	4.65	0.0032	36	3.65	0.0020
with water	0.68	4.59	0.154	0.82	3.49	0.1742

Table S3. **Parameters for fitting the solid surface response to a double-Lorentzian as Eq. S23.**

In Figs. S17, we show the fitted $g_{\mathrm{sol}}(q_0, \omega)$ profile for simulations of the solid with various values of m_{D} in the absence and in the presence of water. While the principal peaks remain at $\omega_0 \approx \omega_1 \approx \omega_{\mathrm{D}}$ in all cases, they begin to broaden and decrease in height more significantly for cases in the strong-coupling regime.

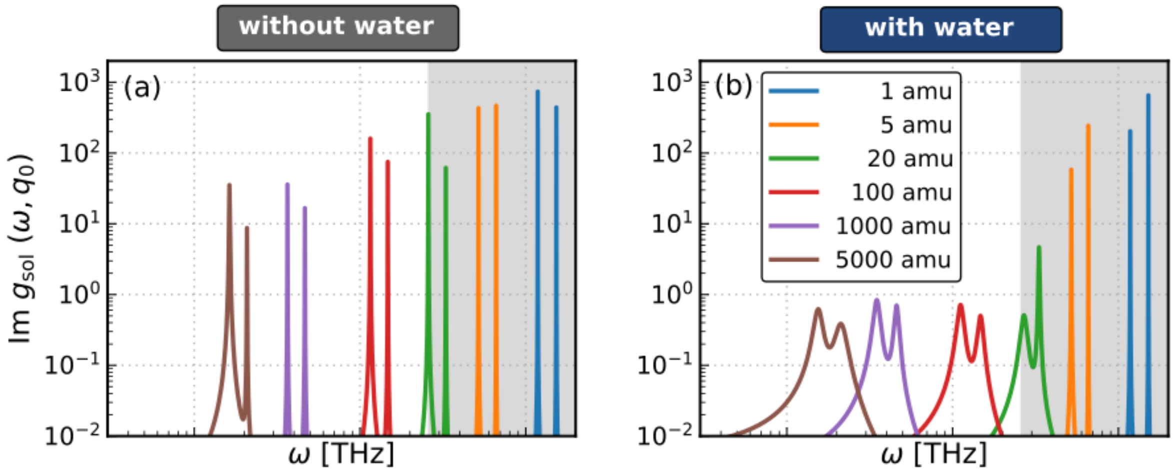

Figure S17. **Solid peaks broaden in the presence of water.** The solid surface response function fitted to a double-Lorentzian function for a range of different m_D (as indicated in the legend) in the (a) absence and (b) presence of water.

S5.3. Water surface response function

For the water surface response in the weak-coupling regime, we focus on $g_{wat}(q, \omega)$ obtained from simulations with $m_D = 1$ amu here as shown in Fig. S18(a). However, the result pertains to all simulations with $m_D \lesssim 20$ amu. At the long wavelength limit ($q \to 0$), we see that water shows a sharp peak at $\omega_{lib} \approx 20$ THz coming from the librational modes and a broad feature spanning $10^{-2} - 10^{1}$ THz. The dielectric fluctuations due to intermolecular modes of water in this regime agree well with previous simulations with different interaction potentials.[38] As q is increased, we see a decrease in $g_{wat}(q, \omega)$. Most importantly, in the weak-coupling regime, the water response function appears unperturbed by the presence of the Drude particles at all wavevectors.

In the strong-coupling regime, we focus on $g_{wat}(q, \omega)$ obtained from simulations with $m_D = 5000$ amu. As shown in Fig. S18(b), at the long wavelength limit ($q \to 0$), we see that $g_{wat}(q, \omega)$ is strongly perturbed, indicating that the water and the Drude particles' motions are strongly coupled. As q increases above $q \approx 1.5\,\text{Å}^{-1}$, this coupling becomes less significant, which is consistent with $g_{sol}(q, \omega)$ decays for $q \gtrsim 1.5\,\text{Å}^{-1}$.

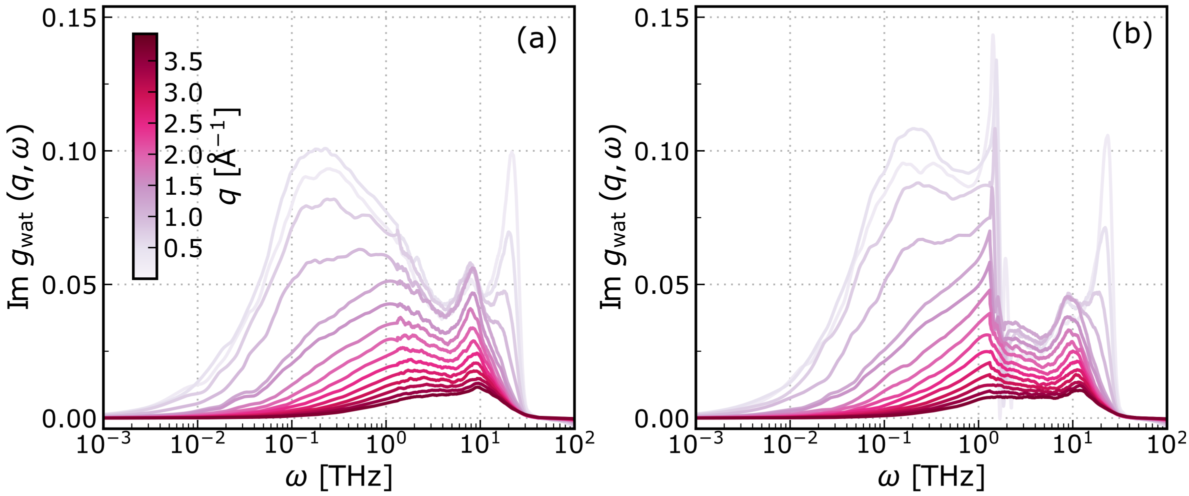

Figure S18. **Water surface response function.** $g_{wat}(q, \omega)$ obtained from simulations (a) in the weak-coupling regime appears unperturbed by the presence of the Drude oscillators. (b) In the strong-coupling regime, $g_{wat}(q, \omega)$ is strongly perturbed by the solid up to $q \approx 1.5\,\text{Å}^{-1}$.

S5.4. Force spectra

To further illustrate the change from the weak-coupling to the strong-coupling regime, we also computed the spectrum of the lateral force defined as

$$S_{\mathrm{F}}(\omega) = \int_{-\infty}^{+\infty} \mathrm{d}t \, \langle \mathcal{F}(0)\mathcal{F}(t) \rangle \, e^{i\omega t}. \tag{S24}$$

We show $S_{\mathrm{F}}(\omega)$ for simulations with Drude masses used the previous subsection in conjunction with the Green–Kubo friction $\lambda_{\mathrm{GK}}(\tau)$ in Fig. S19. For cases belonging to the weak-coupling regime ($m_{\mathrm{D}}/\mathrm{amu} = 1, 2, \text{ and } 5$), $S_{\mathrm{F}}(\omega)$ shows a peak due to the solid charge density at $\omega \approx \omega_0$ determined from $S_{\mathrm{sol}}(q_0, \omega)$, and a broad feature at low ω from the water charge density. As m_{D} is increased, the peak due to the solid starts to merge with the water broad feature and increase in intensity. This can be linked to the behavior of $\lambda_{\mathrm{GK}}(\tau)$ in the strong-coupling regime ($m_{\mathrm{D}}/\mathrm{amu} = 20, 50, 200, 1000 \text{ and } 5000$): the increase in $S_{\mathrm{F}}(\omega = \omega_0)$ is responsible for stronger oscillations in $\lambda_{\mathrm{GK}}(\tau)$ at shorter times ($\tau < \tau_{\mathrm{F}}$) while the increase in $S_{\mathrm{F}}(\omega \lesssim \omega_0)$ is responsible for a higher plateau value at longer times ($\tau \geq \tau_{\mathrm{F}}$).

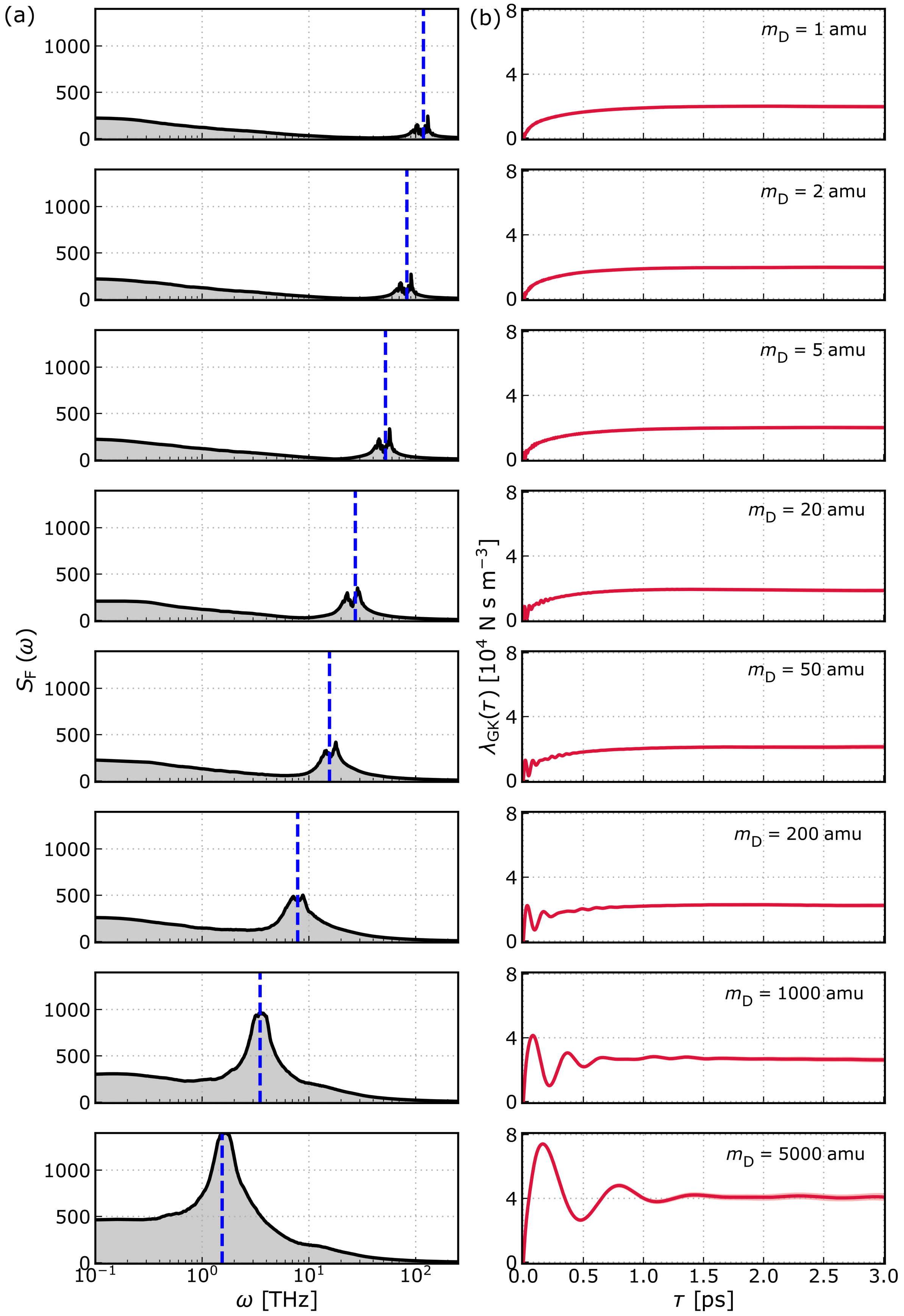

Figure S19. **Lateral force spectra.** (a) $S_F(\omega)$ for simulations with different m_D. The value of ω_0 extracted from $S_{sol}(q_0, \omega)$, marked as a dashed blue line, match with the frequency of the peak in $S_F(\omega)$ due to solid charge density contribution. This peak shows higher intensity as we move from the weak-coupling regime ($m_D < 20$ amu) to the strong-coupling regime ($m_D \geq 20$ amu). (b) This change manifests in $\lambda_{GK}(\tau)$ as the appearance of small oscillations at short τ and the increase in the plateau value at longer τ.

S6. COMPARISON TO QUANTUM FRICTION THEORY

S6.1. Quantum friction formula

In quantum friction theory,[38] Kavokine *et al.* derived an expression for the QF coefficient given as

$$\lambda_{\mathrm{Q}} = \frac{\hbar^2}{8\pi^2 k_{\mathrm{B}} T} \int_0^\infty \mathrm{d}q\, q^3 \int_0^\infty \mathrm{d}\omega\, \frac{1}{\sinh^2(\hbar\omega/2k_{\mathrm{B}}T)} \frac{\operatorname{Im} g_{\mathrm{sol}}(q,\omega) \operatorname{Im} g_{\mathrm{wat}}(q,\omega)}{|1 - g_{\mathrm{sol}}(q,\omega)\, g_{\mathrm{wat}}(q,\omega)|^2}, \tag{S25}$$

where $g_{\mathrm{sol}}(q,\omega)$ and $g_{\mathrm{wat}}(q,\omega)$ are the surface response functions for the solid and water in the absence of any coupling respectively.

S6.2. Water surface response

To evaluate λ_{Q} quantitatively, one needs to obtain $g_{\mathrm{sol}}(q,\omega)$ and $g_{\mathrm{wat}}(q,\omega)$. As in Ref. 38, the water dielectric function is represented by a sum of two Debye peaks at $\omega_{\mathrm{Db},1}$ and $\omega_{\mathrm{Db},2}$, each with an exponentially decaying q dependence

$$g_{\mathrm{wat}}(q,\omega) = \frac{g_{\mathrm{wat}}(q,0)}{2} \left(\frac{\mathrm{e}^{-q/q_0}}{1 - i\omega/\omega_{\mathrm{Db},1}} + \frac{2 - \mathrm{e}^{-q/q_0}}{1 - i\omega/\omega_{\mathrm{Db},2}} \right), \tag{S26}$$

where $g_{\mathrm{wat}}(q,\omega)$ is given as

$$g_{\mathrm{wat}}(q,0) = \mathrm{e}^{a + b[1+(q/c)^d]^{1/d}}. \tag{S27}$$

We have used the same parameters as Ref. 38, which for completeness, are reproduced in Table S4.

$g_{\mathrm{wat}}(q,\omega)$	Werder $g_{\mathrm{wat}}(q,0)$	Aluru $g_{\mathrm{wat}}(q,0)$
$q_0 = 3.12\,\text{Å}^{-1}$	$a = 5.16$	$a = 3.38$
$\omega_{\mathrm{Db},1} = 0.36\,\mathrm{THz}$	$b = -5.19$	$b = -3.41$
$\omega_{\mathrm{Db},2} = 4.84\,\mathrm{THz}$	$c = 1.95\,\text{Å}^{-1}$	$c = 1.79\,\text{Å}^{-1}$
	$d = 2$	$d = 2.4$

Table S4. **Parameters for the analytical expression for the water surface response function in the absence of charge density coupling from the solid.**

For the solid dielectric fluctuations, two different models were used to represent the graphite's dispersionless surface plasmon. Numerical evaluation in Ref. 38 of the QF coefficient using the Aluru $g_{\mathrm{wat}}(q,\omega)$ gave a contribution of $\lambda_{\mathrm{Q}} \approx 0.5 \times 10^4\,\mathrm{N\,s\,m^{-3}}$ with a "Drude" model for the surface plasmon, which is comparable to λ_{THz} obtained from our simulations.

S6.3. Solid surface response with reparameterized Drude model

Encouraged by the agreement between λ_{THz} obtained from simulations and λ_{Q} predicted from QF theory, we can further assess how well our simulations are capturing QF by reparameterizing the Drude model to approximately represent the surface response function of our simple model. In the Drude model for a surface plasmon, which is based on the semi-classical treatment of free electron dynamics,[39] the solid surface response function is of the form

$$g_{\mathrm{sol}}(q,\omega) = \frac{\omega_{\mathrm{p}}^2}{\omega_{\mathrm{p}}^2 - \omega^2 - 2i\eta\,\omega} \Theta(q_{\mathrm{max}} - q), \tag{S28}$$

where ω_{p} is the principal peak due to the plasmon, η is the surface plasmon width, Θ is the Heaviside step function and q_{max} is the cut-off wavevector.

Drawing a parallel mapping to the surface response function obtained in our simulations, we can parameterize the Drude model to represent a surface plasmon with frequency $\omega_p = \omega_0$ corresponding to the principal tangential mode in simulations. We choose $\eta = \omega_0/100$ and $q_{max} = 1.5\,\text{Å}^{-1}$, giving $\mathrm{Im}\, g_{sol}(q < 1.5\,\text{Å}^{-1}, \omega = \omega_p) = 50$, such that the plasmon has a small width and long flat dispersion, and its surface response function matches reasonably well with that obtained in simulations (Fig. S16).

S6.4. Comparison of the quantum friction coefficient

Using the reparameterized Drude model for the solid surface response function and the Werder water surface response function, we evaluated the QF coefficient λ_Q from Eq. S25. For a plasmon mode with $\omega_p = 10\,\text{THz}$, the reparameterized Drude model gives $\lambda_Q \approx 0.2 \times 10^4\,\text{N s m}^{-3}$. This is close to the value obtained with the Drude model in Ref. 38 and the quantitative difference can be explained by comparing the contribution of the integrand of Eq. S25, $\tilde{\lambda}_Q$, in (q, ω) space, as shown in Fig. S20.

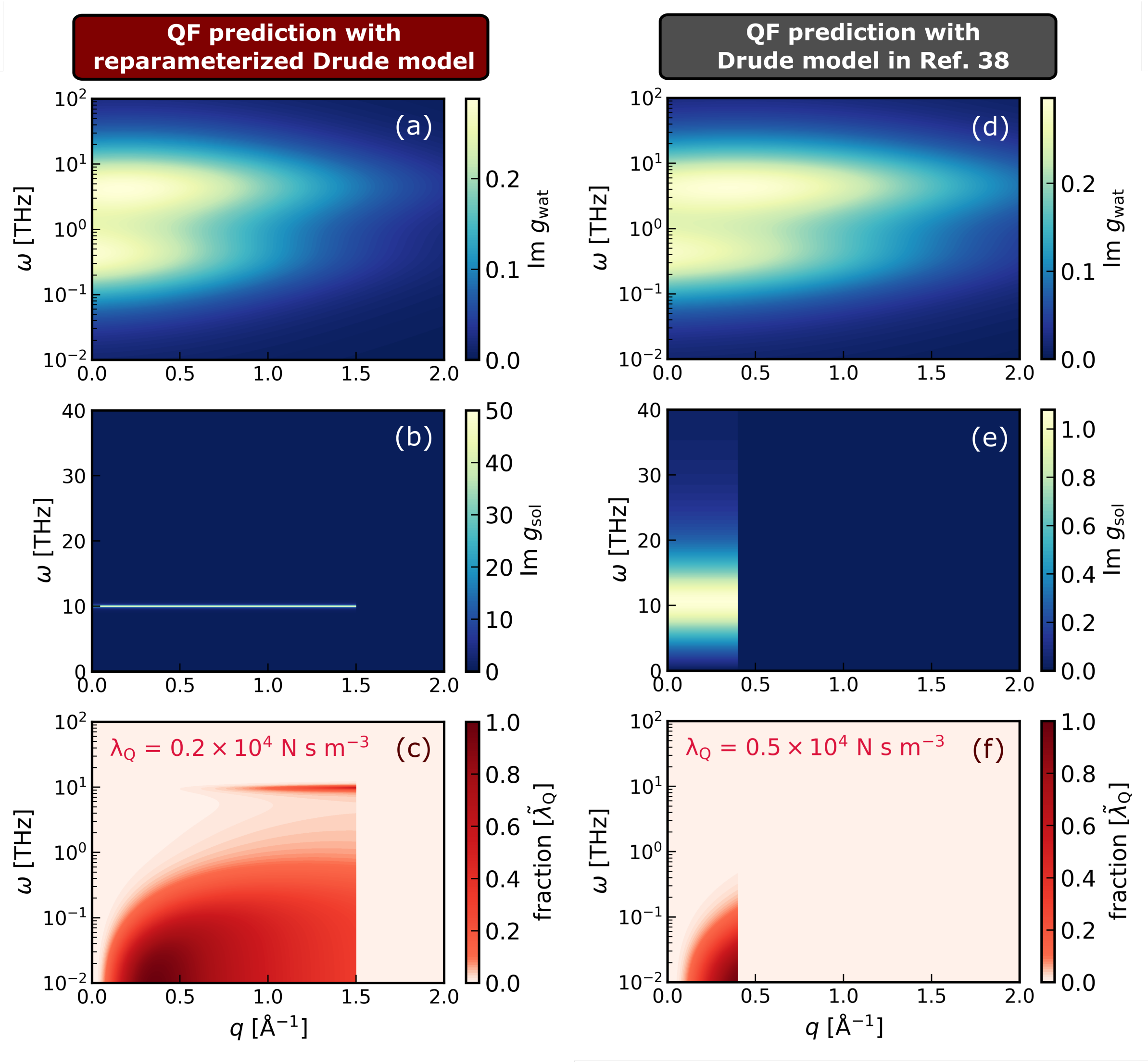

Figure S20. **Comparison of the quantum friction coefficient with parameters from simulations (a-c) and from Ref. 38 (d-f).** (a) The water Werder surface response function. (b) The solid surface response function from the reparameterized Drude model. (c) The QF integrand contributes mainly at ($\omega \lesssim 1\,\text{THz}$, $q < 1.5\,\text{Å}^{-1}$) and at ($\omega \approx \omega_p = 10\,\text{THz}$, $1 < q/\text{Å}^{-1} < 1.5$). (d) The water Aluru surface response function. (e) The solid surface response function from the Drude model with parameters from Ref. 38. (f) The QF integrand contributes mainly at ($\omega \lesssim 0.1\,\text{THz}$, $q < 0.5\,\text{Å}^{-1}$).

Since there is little difference between the water surface response functions $g_{\mathrm{wat}}(q,\omega)$ in the two cases, the difference comes down to the solid surface response $g_{\mathrm{sol}}(q,\omega)$ or ultimately the plasmon dispersion. Since the plasmon in the reparameterized Drude model spans to a higher wavevector compared to the Drude model in Ref. 38, $\tilde{\lambda}_{\mathrm{Q}}$ contributes significantly to the final integral not only at ($\omega \lesssim 1\,\mathrm{THz}$, $q < 1.5\,\mathrm{\AA}^{-1}$) but also at ($\omega \approx \omega_{\mathrm{p}} = 10\,\mathrm{THz}$, $1 < q/\mathrm{\AA}^{-1} < 1.5$). However, since the width of the plasmon in the reparameterized Drude model is much smaller, the final integral value for λ_{Q} is still smaller.

S6.5. Sensitivity of the dependence of quantum friction on the solid frequency

From QF theory, we can also map out the dependence of the QF friction coefficient as a function of frequency of the solid mode. Using the reparameterized Drude model, as presented in the main article, we see a very good agreement between λ_{Q} calculated from theory and λ_{THz} obtained from simulations in the frequency range where graphite surface plasmons are experimentally observed.

In the Drude model, both the width η and the wavevector cut-off q_{max} control the plasmon dispersion and changing their values will affect the solid surface response function $g_{\mathrm{sol}}(q,\omega)$ and therefore the friction. We can also check the sensitivity of the dependence of the QF coefficient on the plasmon frequency upon changing to different values for each of these parameters. In simulations with the solid modes' frequencies in the range $2-20\,\mathrm{THz}$ where graphite's surface plasmons are experimentally observed, the solid modes' wavevector cut-offs can sensibly range between $q_{\mathrm{max}} \approx 1.4-2.0\,\mathrm{\AA}^{-1}$ and their amplitudes can sensibly range between $\mathrm{Im}\,g_{\mathrm{sol}}(q < q_{\mathrm{max}}, \omega = \omega_{\mathrm{p}}) \approx 30-100$. This corresponds to $\eta/\omega_0 \approx 1/60 - 1/200$ in the Drude model. In Fig. S21(a), we show the dependence of QF on ω_0 using the Drude model for different values of q_{max} while keeping the width at $\eta = \omega_0/100$. As q_{max} increases, there is a higher contribution to the QF integral at higher q, leading to a higher λ_{Q}. In Fig. S21(b), we show again this dependence for different values of the width η/ω_0 while while keeping the wavevector cut-off at $q_{\mathrm{max}} = 1.5\,\mathrm{\AA}^{-1}$. As η/ω_0 increases, the width of the plasmon decreases, leading to lower values of λ_{Q}. In all cases, however, there is only a significant contribution to QF when $\omega_0 \lesssim 20\,\mathrm{THz}$, which agrees with the results in the strong-coupling regime from simulations.

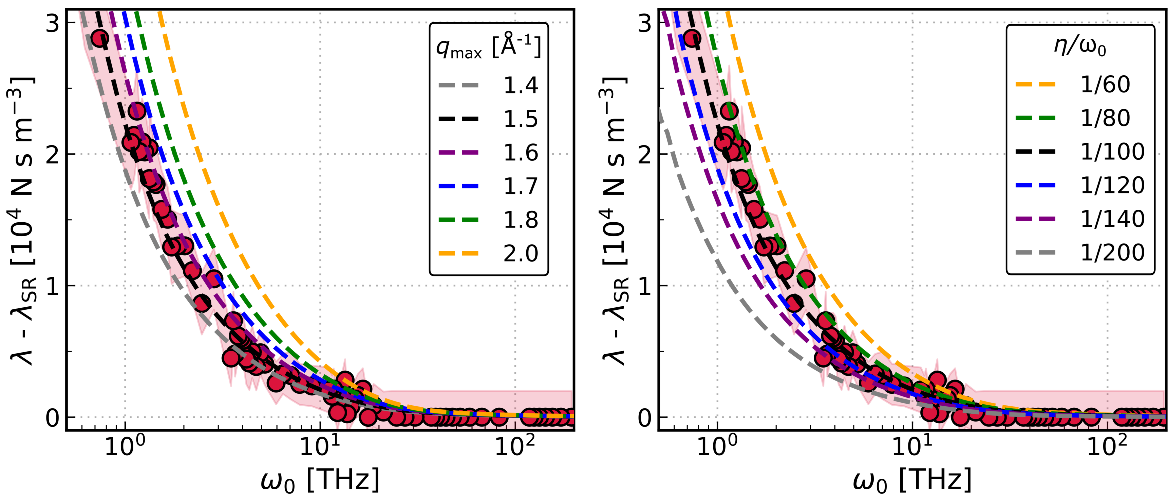

Figure S21. **Dependence of the quantum friction coefficient on the solid charge density frequency from the Drude model** (a) for different values of q_{max} (indicated in the legend) while keeping $\eta = \omega_0/100$ and (b) for different values of η/ω keeping $q_{\mathrm{max}} = 1.5\,\mathrm{\AA}^{-1}$. The calculated λ_{Q} from theory is shown with dashed lines while λ_{THz} extracted from simulations are shown as red points with the shaded red region as the error from block-averaging.

S7. ADDITIONAL PROPERTIES OF THE INTERFACE

S7.1. Static properties

For static properties in the liquid, we analyse the density profiles of the water along the surface normal. These are identical for the weak-coupling and the strong-coupling cases, as shown in Fig. S22(a). In the solid, the magnitude of the dipole moment of a Drude oscillator can be obtained from $\mu_{\mathrm{D}} = Q_{\mathrm{D}}d$ where d is the distance of the Drude particle from the core atom. We look at its probability distribution $p(\mu_D)$ for both cases, as shown in Fig. S22(b). In both cases, $p(\mu_D)$ is identical, reinforcing that the interatomic potential, and therefore all static equilibrium properties of the interface are not affected when m_{D} is varied.

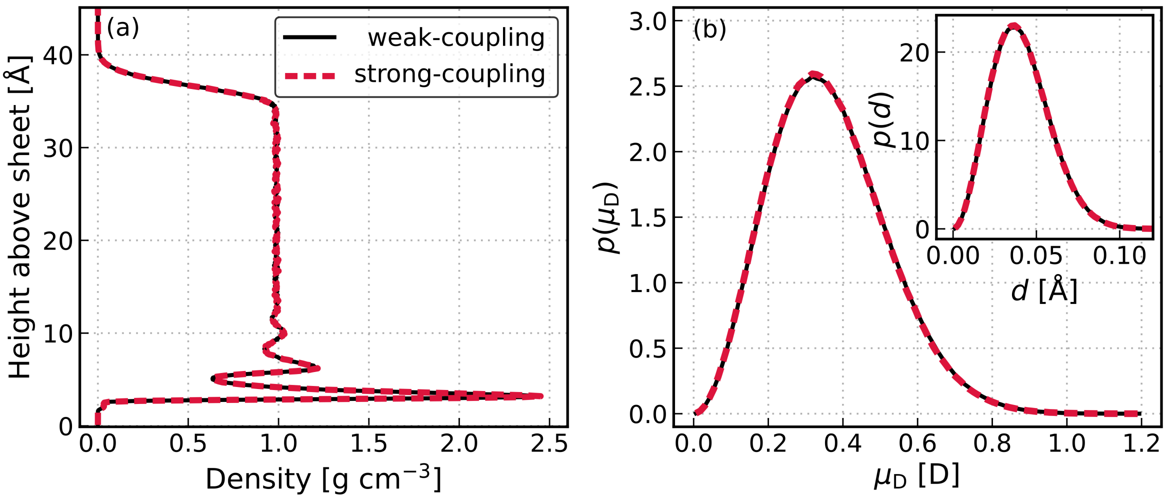

Figure S22. **Static equilibrium properties.** (a) The planar mass density profiles of the water are identical for the weak-coupling and the strong-coupling cases, showing a maximum density of $\approx 2.5\,\mathrm{g\,cm^{-3}}$ and is at a height ≈ 3.2 Å for the contact layer of water. (b) The probability distributions of the dipole moment magnitude of a Drude oscillator $p(\mu_{\mathrm{D}})$ in the solid are also identical for both cases, with the average of the distribution at $\langle \mu_{\mathrm{D}} \rangle \approx 0.36$ D. The inset shows the distribution of the distance between the Drude particles and their cores $p(d)$.

To link these observations to the unchanged static component of the friction coefficient described in the main article, we also look at the corrugation of the free energy surface (FES) experienced by the water molecules. Following previous work,[22,40,41] the two-dimensional FES of species i is given by

$$\Delta G_i(x,y) = -k_{\mathrm{B}}T \ln[p_i(x,y)], \quad \text{(S29)}$$

where $p_i(x,y)$ is the normalized two-dimensional probability of finding species i in the contact layer at point (x,y). For every saved configuration, we define the contact layer as consist of water molecules with height above the sheet ≤ 5 Å, where the first minimum in the density profile is. After computing both $p_{\mathrm{O}}(x,y)$ and $p_{\mathrm{H}}(x,y)$ and averaging onto a unit cell in the solid, we obtained the oxygen-based and hydrogen-based FESs. To ensure each surface is independent of noise, a Savitzky–Golay filter[25] was applied. The corrugation of each FES, ΔG_{O} and ΔG_{H}, can be quantified by taking the highest free energy present in the FES. As shown in Fig. S23, the water molecules experience the same oxygen-based and hydrogen-based FES at the liquid–solid interface in the weak-coupling and the strong-coupling regimes. Since corrugation is much more pronounced in the oxygen-based FES, we show ΔG_{O} as an approximation of the total corrugation in the main article.

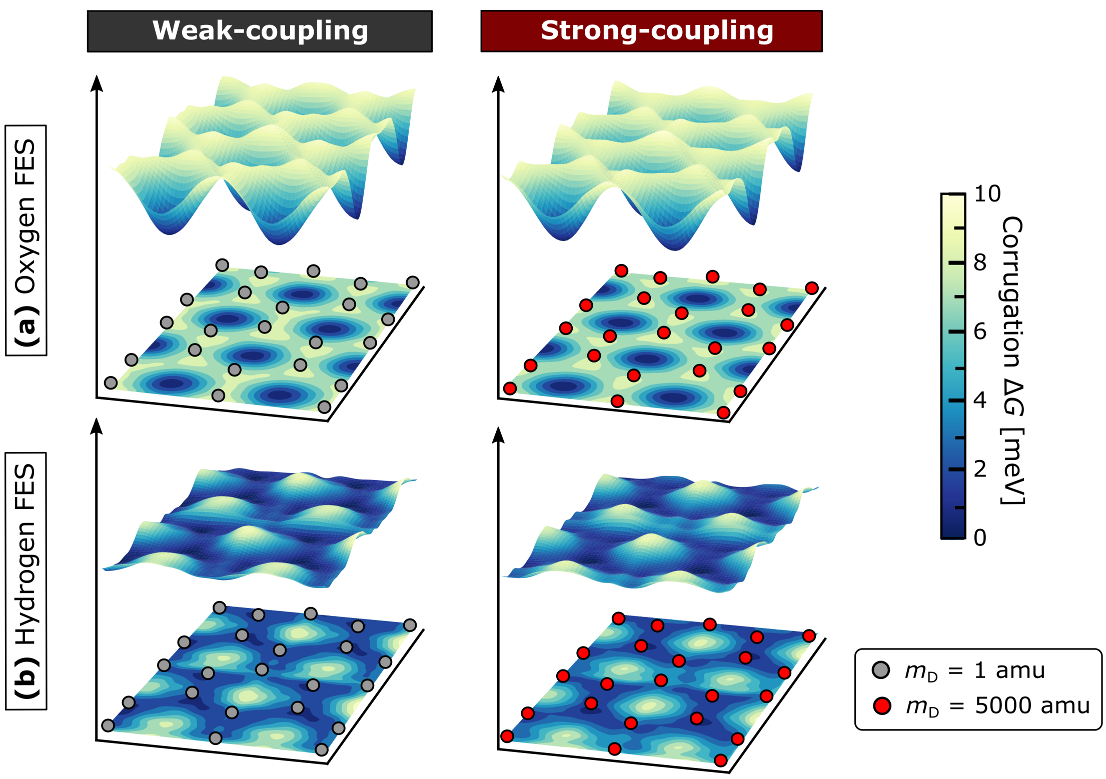

Figure S23. **Free energy surface corrugation** (a) The oxygen-based FES shows that oxygen atoms preferentially sit on hollow sites in the middle of the hexagon rings in graphene. (b) The hydrogen-based FES, which is less corrugated than the oxygen-based FES, shows that hydrogen atoms preferentially sit on carbon sites. The solid atoms are represented by the markers in the projection where the core atom in the Drude oscillator with $m_D = 1\,\mathrm{amu}$ is in grey and $m_D = 5000\,\mathrm{amu}$ in red. Both FESs show identical level of corrugation in the weak-coupling and strong-coupling cases.

S7.2. Charge density relaxation in the water film

Instead of looking at the charge density relaxation at just the surface, we can also characterize the relaxation of the whole water film. We can define the Fourier components of the charge densities for the solid and the liquid as

$$\tilde{n}_{\mathrm{sol}}(q,t) = \sum_{\alpha\,\in\,\mathrm{sol}} Q_\alpha e^{i\boldsymbol{q}\cdot\boldsymbol{x}_\alpha(t)}, \tag{S30}$$

$$\tilde{n}_{\mathrm{wat}}(q,t) = \sum_{\alpha\,\in\,\mathrm{wat}} Q_\alpha e^{i\boldsymbol{q}\cdot\boldsymbol{x}_\alpha(t)}, \tag{S31}$$

where we have implicitly only considered the zero wavevector in the direction normal to the graphene sheet. We can again characterize the relaxation of these charge densities with the following autocorrelation functions

$$C_{\mathrm{sol}}(\tau;q) = \frac{\langle \tilde{n}_{\mathrm{sol}}(q,0)\,\tilde{n}_{\mathrm{sol}}(-q,\tau)\rangle}{\langle |\tilde{n}_{\mathrm{sol}}(q)|^2\rangle}, \tag{S32}$$

$$C_{\mathrm{wat}}(\tau;q) = \frac{\langle \tilde{n}_{\mathrm{wat}}(q,0)\,\tilde{n}_{\mathrm{wat}}(-q,\tau)\rangle}{\langle |\tilde{n}_{\mathrm{wat}}(q)|^2\rangle}. \tag{S33}$$

Focusing on the long-wavelength limit, we show the results for $C_{\mathrm{sol}}(\tau;q_0)$ and $C_{\mathrm{wat}}(\tau;q_0)$ in Fig. S24. Again, the solid modes relax on a much faster timescale in the strong- than in the weak-coupling regime.

The water relaxation, however, barely differs between the two regimes, meaning any response in the liquid due to coupling with the Drude motions is localized the surface and does not affect the response of the whole film of water significantly as a whole.

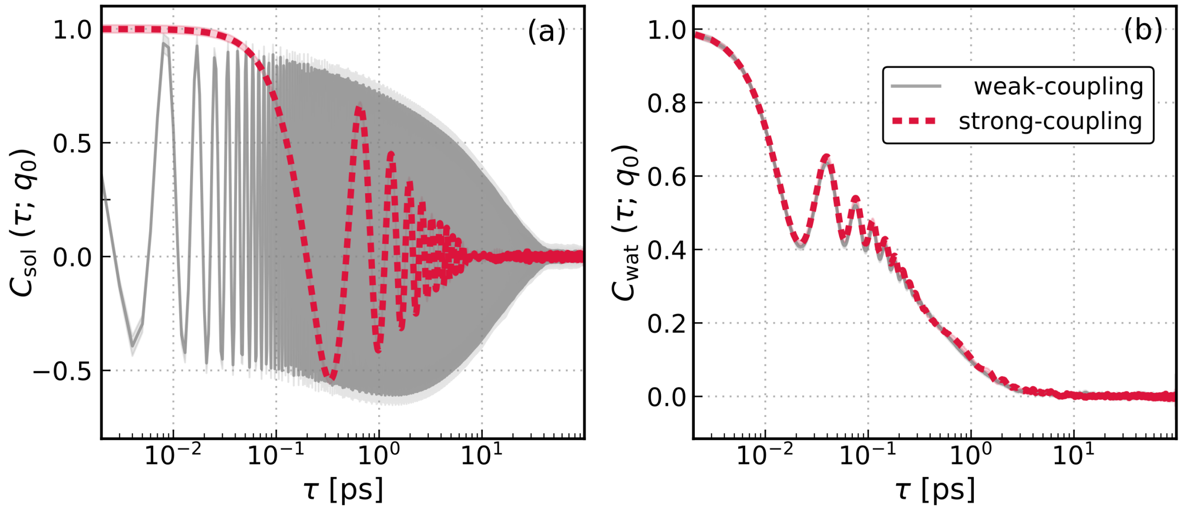

Figure S24. **Charge density correlation: asymmetry in response between the liquid and the solid.** (a) The relaxation of the solid charge density is much faster in the strong-coupling than the weak-coupling regime while (b) relaxation of the charge density of the whole water film barely differs between the two regimes.

S7.3. Other dynamical properties

In addition to the charge density relaxation, we also explored other dynamical properties of water including its orientational relaxation and hydrogen-bonding relaxation. The orientational dynamics of water molecules in the liquid is examined, following Yeh and Mou,[42] via the second-order rotational autocorrelation function, defined as

$$C_{\mathrm{rot}}(\tau) = \langle P_2[\boldsymbol{u}(0) \cdot \boldsymbol{u}(\tau)] \rangle, \tag{S34}$$

where $\boldsymbol{u}(\tau)$ is the unit vector along the water molecular dipole at time τ and $P_2(x)$ denotes the second Legendre Polynomial. The hydrogen-bond relaxation is examined via the autocorrelation function of the presence of a hydrogen bond, defined as

$$C_{\mathrm{hb}}(\tau) = \frac{\langle h(0) \cdot h(\tau) \rangle}{\langle h^2 \rangle}, \tag{S35}$$

where $h(\tau) = 1$ if there is a hydrogen bond between a pair of water molecules at time τ and $h(\tau) = 0$ otherwise. Two water molecules are considered to be hydrogen-bonded according to geometric criteria from Luzar and Chandler.[43]

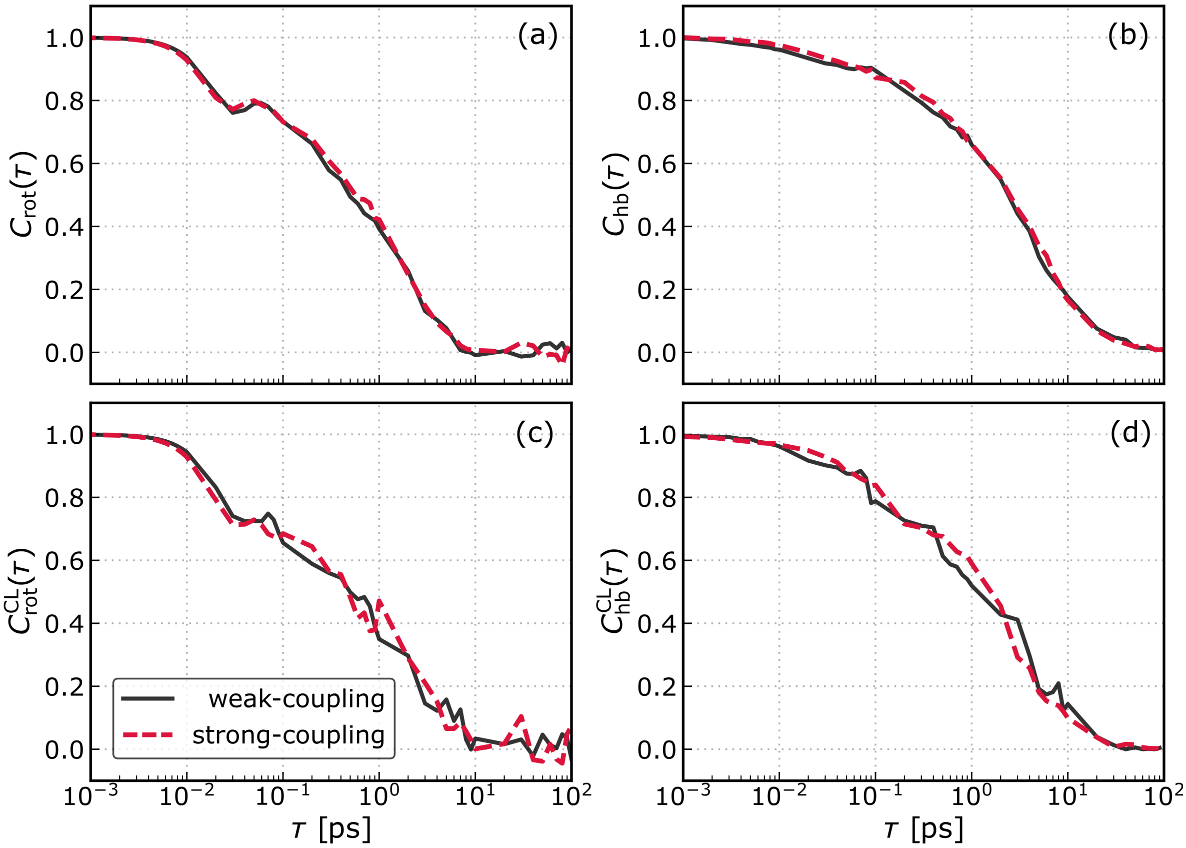

Figure S25. **Dynamical properties of water.** The rotational autocorrelation (a and c) and the hydrogen-bonding autocorrelation (b and d) are both barely affected between the weak-coupling and the strong-coupling regimes. The functions are computed for the whole water film (a and b) and just the contact layer (c and d).

From Figs. S25(a) and (b), we see little differences between the weak-coupling and strong-coupling cases for both $C_{\mathrm{rot}}(\tau)$ and $C_{\mathrm{hb}}(\tau)$ computed for the whole water film. These observations still hold when we look at $C_{\mathrm{rot}}^{\mathrm{CL}}(\tau)$ and $C_{\mathrm{hb}}^{\mathrm{CL}}(\tau)$, in Figs. S25(c) and (d), where superscript CL denotes that the autocorrelation functions are defined for just the contact layer (defined as the layer from the carbon sheet up to the first minimum of the water density profile). This supports our conclusion that the increase in friction due to charge density coupling has little impact on local dynamical properties of the liquid.

REFERENCES

[1] G. Lamoureux and B. Roux, "Modeling induced polarization with classical Drude oscillators: Theory and molecular dynamics simulation algorithm," The Journal of Chemical Physics **119**, 3025–3039 (2003).

[2] B. T. Thole, "Molecular polarizabilities calculated with a modified dipole interaction," Chemical Physics **59**, 341–350 (1981).

[3] C. Schröder and O. Steinhauser, "Simulating polarizable molecular ionic liquids with Drude oscillators," The Journal of Chemical Physics **133**, 154511 (2010).

[4] S. Y. Noskov, G. Lamoureux, and B. Roux, "Molecular dynamics study of hydration in ethanolwater mixtures using a polarizable force field," The Journal of Physical Chemistry B **109**, 6705–6713 (2005).

[5] G. Lamoureux, E. Harder, I. V. Vorobyov, B. Roux, and A. D. MacKerell, "A polarizable model of water for molecular dynamics simulations of biomolecules," Chemical Physics Letters **418**, 245–249 (2006).

[6] J.-L. Barrat and L. Bocquet, "Influence of wetting properties on hydrodynamic boundary conditions at a fluid/solid interface," Faraday Discuss. **112**, 119–128 (1999).

[7] K. Falk, F. Sedlmeier, L. Joly, R. R. Netz, and L. Bocquet, "Ultralow liquid/solid friction in carbon nanotubes: Comprehensive theory for alcohols, alkanes, OMCTS, and water," Langmuir **28**, 14261–14272 (2012).

[8] S. Plimpton, "Fast parallel algorithms for short-range molecular dynamics," Journal of Computational Physics **117**, 1–19 (1995).

[9] A. P. Thompson, H. M. Aktulga, R. Berger, D. S. Bolintineanu, W. M. Brown, P. S. Crozier, P. J. in 't Veld, A. Kohlmeyer, S. G. Moore, T. D. Nguyen, R. Shan, M. J. Stevens, J. Tranchida, C. Trott, and S. J. Plimpton, "LAMMPS - a flexible simulation tool for particle-based materials modeling at the atomic, meso, and continuum scales," Computer Physics Communications **271**, 108171 (2022).

[10] H. J. C. Berendsen, J. R. Grigera, and T. P. Straatsma, "The missing term in effective pair potentials," The Journal of Physical Chemistry **91**, 6269–6271 (1987).

[11] H. C. Andersen, "Rattle: A "velocity" version of the shake algorithm for molecular dynamics calculations," Journal of Computational Physics **52**, 24–34 (1983).

[12] T. Werder, J. H. Walther, R. L. Jaffe, T. Halicioglu, and P. Koumoutsakos, "On the water–carbon interaction for use in molecular dynamics simulations of graphite and carbon nanotubes," The Journal of Physical Chemistry B **107**, 1345–1352 (2003).

[13] A. Dequidt, J. Devémy, and A. A. H. Prádua, "Thermalized Drude oscillators with the LAMMPS molecular dynamics simulator," Journal of Chemical Information and Modeling **56**, 260–268 (2016).

[14] R. P. Misra and D. Blankschtein, "Insights on the role of many-body polarization effects in the wetting of graphitic surfaces by water," The Journal of Physical Chemistry C **121**, 28166–28179 (2017).

[15] R. Hockney and J. Eastwood, *Computer Simulation Using Particles* (Adam-Hilger, 1988).

[16] J. Kolafa and J. W. Perram, "Cutoff errors in the ewald summation formulae for point charge systems," Molecular Simulation **9**, 351–368 (1992).

[17] W. Shinoda, M. Shiga, and M. Mikami, "Rapid estimation of elastic constants by molecular dynamics simulation under constant stress," Phys. Rev. B **69**, 134103 (2004).

[18] M. E. Tuckerman, J. Alejandre, R. López-Rendón, A. L. Jochim, and G. J. Martyna, "A liouville-operator derived measure-preserving integrator for molecular dynamics simulations in the isothermal–isobaric ensemble," Journal of Physics A: Mathematical and General **39**, 5629–5651 (2006).

[19] L. Bocquet and J.-L. Barrat, "Hydrodynamic boundary conditions, correlation functions, and kubo relations for confined fluids," Phys. Rev. E **49**, 3079–3092 (1994).

[20] L. Bocquet, J.-P. Hansen, and J. Piasecki, "Friction tensor for a pair of brownian particles: Spurious finite-size effects and molecular dynamics estimates," Journal of Statistical Physics **89**, 321–346 (1997).

[21] P. Español, J. A. de la Torre, and D. Duque-Zumajo, "Solution to the plateau problem in the Green–Kubo formula," Phys. Rev. E **99**, 022126 (2019).

[22] G. Tocci, L. Joly, and A. Michaelides, "Friction of water on graphene and hexagonal boron nitride from ab initio methods: Very different slippage despite very similar interface structures," Nano Letters **14**, 6872–6877 (2014).

[23] A. R. Poggioli and D. T. Limmer, "Distinct chemistries explain decoupling of slip and wettability in atomically smooth aqueous interfaces," The Journal of Physical Chemistry Letters **12**, 9060–9067 (2021).

[24] H. Oga, Y. Yamaguchi, T. Omori, S. Merabia, and L. Joly, "Green–Kubo measurement of liquid-solid friction in finite-size systems," The Journal of Chemical Physics **151**, 054502 (2019).

[25] A. Savitzky and M. J. E. Golay, "Smoothing and differentiation of data by simplified least squares procedures." Analytical Chemistry **36**, 1627–1639 (1964).

[26] A. Seal and A. Govind Rajan, "Modulating water slip using atomic-scale defects: Friction on realistic hexagonal boron nitride surfaces," Nano Letters **21**, 8008–8016 (2021).

[27] L. Joly, G. Tocci, S. Merabia, and A. Michaelides, "Strong coupling between nanofluidic transport and interfacial chemistry: How defect reactivity controls liquid-solid friction through hydrogen bonding," The Journal of Physical Chemistry Letters **7**, 1381–1386 (2016).

[28] C. Rupakheti, G. Lamoureux, A. D. MacKerell, and B. Roux, "Statistical mechanics of polarizable force fields based on classical drude oscillators with dynamical propagation by the dual-thermostat extended lagrangian," The Journal of Chemical Physics **153**, 114108 (2020).

[29] G. Bussi, D. Donadio, and M. Parrinello, "Canonical sampling through velocity rescaling," The Journal of Chemical Physics **126**, 014101 (2007).

[30] J. C. Shelly, "Boundary condition effects in simulations of water confined between planar walls," Molecular Physics **88**, 385–398 (1996).

[31] J. Alejandre, D. J. Tildesley, and G. A. Chapela, "Molecular dynamics simulation of the orthobaric densities and surface

tension of water," The Journal of Chemical Physics **102**, 4574–4583 (1995).

[32] S. E. Feller, R. W. Pastor, A. Rojnuckarin, S. Bogusz, and B. R. Brooks, "Effect of electrostatic force truncation on interfacial and transport properties of water," The Journal of Physical Chemistry **100**, 17011–17020 (1996).

[33] C. Zhang and M. Sprik, "Computing the dielectric constant of liquid water at constant dielectric displacement," Phys. Rev. B **93**, 144201 (2016).

[34] C. Zhang and M. Sprik, "Finite field methods for the supercell modeling of charged insulator/electrolyte interfaces," Phys. Rev. B **94**, 245309 (2016).

[35] S. J. Cox and M. Sprik, "Finite field formalism for bulk electrolyte solutions," The Journal of Chemical Physics **151**, 064506 (2019).

[36] I.-C. Yeh and M. L. Berkowitz, "Ewald summation for systems with slab geometry," The Journal of Chemical Physics **111**, 3155–3162 (1999).

[37] Y. Wu, H. L. Tepper, and G. A. Voth, "Flexible simple point-charge water model with improved liquid-state properties," The Journal of Chemical Physics **124**, 024503 (2006).

[38] N. Kavokine, M.-L. Bocquet, and L. Bocquet, "Fluctuation-induced quantum friction in nanoscale water flows," Nature **602**, 84–90 (2022).

[39] J. M. Pitarke, V. M. Silkin, E. V. Chulkov, and P. M. Echenique, "Theory of surface plasmons and surface-plasmon polaritons," Reports on Progress in Physics **70**, 1–87 (2006).

[40] G. Tocci, M. Bilichenko, L. Joly, and M. Iannuzzi, "Ab initio nanofluidics: disentangling the role of the energy landscape and of density correlations on liquid/solid friction," Nanoscale **12**, 10994–11000 (2020).

[41] F. L. Thiemann, C. Schran, P. Rowe, E. A. Müller, and A. Michaelides, "Water flow in single-wall nanotubes: Oxygen makes it slip, hydrogen makes it stick," ACS Nano **16**, 10775–10782 (2022).

[42] Y.-l. Yeh and C.-Y. Mou, "Orientational relaxation dynamics of liquid water studied by molecular dynamics simulation," The Journal of Physical Chemistry B **103**, 3699–3705 (1999).

[43] A. Luzar and D. Chandler, "Hydrogen-bond kinetics in liquid water," Nature **379**, 55–57 (1996).
